# Impact of ivermectin and vector control on onchocerciasis transmission in Togo: Assessing the empirical evidence on trends in infection and entomological indicators

**DOI:** 10.1371/journal.pntd.0012312

**Published:** 2024-07-22

**Authors:** Natalie V. S. Vinkeles Melchers, Sibabi Agoro, Kwamy Togbey, Koffi Padjoudoum, Ibrahim Gado Telou, Potchoziou Karabou, Touka Djatho, Michel Datagni, Ameyo Monique Dorkenoo, Yao Kassankogno, Rachel Bronzan, Wilma A. Stolk

**Affiliations:** 1 Health & Society Group, Department of Social Sciences, Wageningen University & Research, Hollandseweg 1, Wageningen, The Netherlands; 2 Department of Public Health, Erasmus MC, University Medical Center Rotterdam, Rotterdam, The Netherlands; 3 National Institute of Hygiene, Ministry of Health, Public Hygiene and Universal Access to Care, Lomé, Togo; 4 Faculté Des Sciences de La Santé, Université de Lomé, Boulevard Eyadema, Lomé, Togo; 5 Health and Development International (HDI), Lomé, Togo; 6 Bill & Melinda Gates Foundation, Seattle, Washington, United States of America; Seoul National University College of Medicine, REPUBLIC OF KOREA

## Abstract

**Background:**

The World Health Organization’s 2021–2030 Road Map for Neglected Tropical Diseases boosted global commitment towards the elimination of onchocerciasis, but the timeline to elimination will vary strongly between countries in Africa. To assess progress towards elimination in the Republic of Togo, we reviewed the history of control and time trends in infection.

**Methodology/principal findings:**

We collated all available programmatic, entomological, and epidemiological data since the initiation of the Onchocerciasis Control Programme (OCP) in Togo through different data sources. We then visualised data trends over time, to assess the impact of interventions on infection and transmission levels. Vector control was initiated by OCP from 1977 (northern and central parts of Togo) or 1988 (southern regions) up to 2002 (most areas) or 2007 (“special intervention zones” [SIZ], parts of Northern and Central Togo). Between 1988 and 1991, Togo initiated ivermectin mass drug administration (MDA) in eligible communities. The impact of vector control was high in most river basins, resulting in low annual biting rates and annual transmission potential declining to very low levels; the impact was lower in river basins designated as SIZ. Repeated, longitudinal ivermectin mass treatments have overall strongly reduced onchocerciasis transmission in Togo. Epidemiological surveys performed between 2014 and 2017 showed that the prevalence of skin microfilariae (mf) and anti-OV16 IgG4 antibodies had declined to low levels in several districts of the Centrale, Plateaux, and Maritime region. Yet, relatively high mf prevalences (between 5.0% and 32.7%) were still found in other districts during the same period, particularly along the Kéran, Mô and Ôti river basins (SIZ areas).

**Conclusions/significance:**

Trends in infection prevalence and intensity show that onchocerciasis levels have dropped greatly over time in most areas. This demonstrates the large impact of long-term and wide-scale interventions, and suggest that several districts of Togo are approaching elimination.

## Author summary

Onchocerciasis (river blindness) is a vector-borne parasitic disease, which can cause stigmatising and disabling skin and eye disease. Throughout Africa, mass drug administration (MDA) with ivermectin and vector control have effectively reduced and prevented the spread of infection. The Onchocerciasis Control Programme (1974–2002) has implemented long-term and large-scale interventions in West-Africa to control onchocerciasis. However, there are still reports of onchocerciasis transmission in many areas. In the Republic of Togo, the northern and central parts were initially most endemic, particularly high infection prevalence around the Ôti, Kéran and Mô river basins. To support policy making towards elimination, we reviewed transmission conditions, the history of control, and trends in various infection indicators over time in Togo from the start of interventions to 2018. We assessed that vector control and ivermectin MDA impacted levels of infection prevalence throughout the country, with over 88% of surveyed villages since 2012 having overall onchocerciasis prevalence below 5%. Some areas would likely need additional or intensified interventions to bring down transmission, in particular communities along the river basins of Amou (Mono), Kara, Kéran, Ôti (Penjari), and Mô.

## Background

Onchocerciasis (river blindness) has known a long history of control in Africa. This vector-borne parasitic disease can cause stigmatising and disabling skin and eye disease (sub-cutaneous nodules, itching, atrophy, hanging groin, depigmentation, visual impairment, blindness [1–3]) and has been associated with epilepsy [4–7]. The World Health Organization (WHO) has targeted onchocerciasis for elimination and the 2021–2030 Road Map for Neglected Tropical Diseases (NTDs) gave new impetus to this effort [8].

In West-Africa, large-scale interventions were first implemented by the Onchocerciasis Control Programme (OCP, 1974–2002), aiming to eliminate the disease as a public health concern and an impediment to socio-economic development. Initially, the programme focussed solely on vector control through aerial spraying of larvicides in riverbeds, where the blackfly vector of onchocerciasis breeds (genus Simulium). This was complemented by ivermectin mass treatment from 1987 onward, following the demonstration of the safety and effectiveness of ivermectin [9–11] and the generous donation of Mectizan by Merck Inc. & Co [12,13]. Ivermectin mass treatment was implemented alongside vector control or as standalone strategy. When OCP closed in 2002, *O*. *volvulus* infection prevalence and intensity levels had greatly declined in a large part of OCP’s target area and onchocerciasis was no longer a major public health concern [14].

In the northern and central parts of Republic of Togo, vector control was initiated by OCP in 1977 (as part of the phase III Eastern Extension of OCP) [15]. The southern regions of Togo were added in 1988 as OCP extension areas, and between 1988 and 1991, most endemic districts of Togo initiated ivermectin distribution. When OCP closed in 2002, infection prevalences were still relatively high (>5%-59%) in the Ôti, Kéran and Mô river basins and these areas were therefore designated as a “special intervention zone” (SIZ). In this SIZ area, aerial larviciding was continued for another five years (2003–2007) and the frequency of ivermectin treatment was increased from annual to semi-annual [16,17]. Semi-annual treatment is still ongoing in the former SIZ areas.

Although decades-long interventions have led to a strong reduction in infection levels, there are still indications of ongoing transmission [18,19]. To support policy making towards elimination, we reviewed transmission conditions, the history of control, and trends in various infection indicators over time in Togo from the start of interventions to date. This has helped to identify areas where elimination is possibly already achieved or where intensified interventions may be needed to achieve elimination by 2030.

## Methods

### Togo

Togo is bordered by Benin in the East, Ghana in the West, and Burkina Faso to the north. The country covers 56,600 km^2^ [20] with a total population of 9.1 million (2024 census). Since the most recent district demarcation in 2021, the country is administratively divided into five regions and 39 districts (formerly 35 then 44 districts, **Section A in S1 Text**) [21]. The northern regions of Togo are characterised by a mostly dry climate and tropical sub-Saharan savanna, while the southern regions experiences more rainfall.

**Fig 1:**
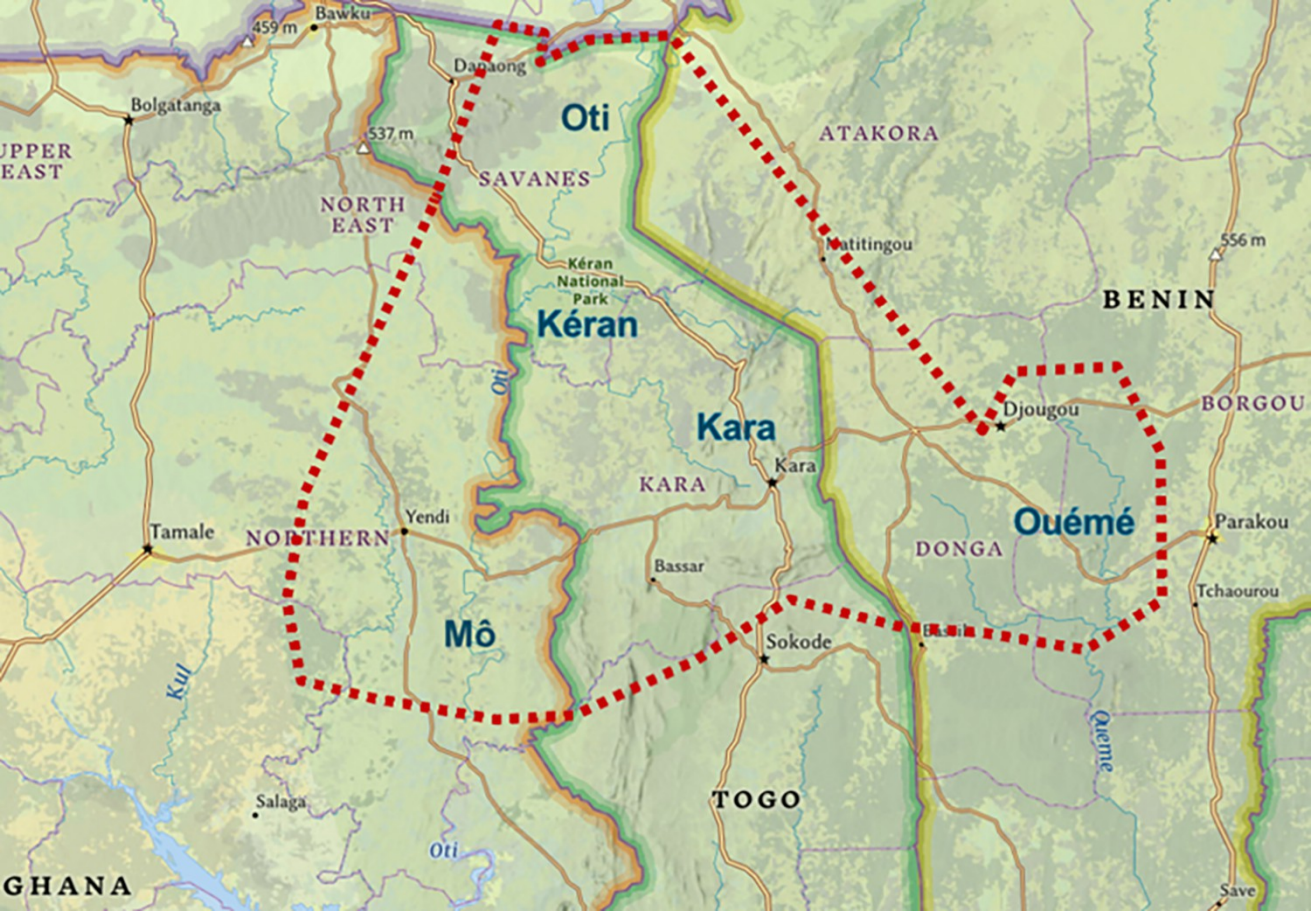
Map showing the major rivers of the Oti Special Intervention Zone, which covers parts of Ghana, Togo, and Benin. In Togo, SIZ areas cover (parts) of the regions Savanes, Kara, and Centrale. The red dotted line are the border lines of the SIZ area. The green and purple shaded line are country borders. The capitalised words indicate regions. The larger dark-blue words (i.e., Oti, Kéran, Kara, Ouémé, Mô) are the respective river basins. *Figure created in ArcGIS Online (**https*:*//www*.*esri*.*com/en-us/arcgis/products/arcgis-online/overview**)*. *All maps are in the public domain*. *Sources*: *Esri*, *USGS | Spatial Solutions & Services*, *Esri*, *HERE*, *Garmin*, *FAO*, *NOAA*, *USGS*. *Link to license information*: *https*:*//doc*.*arcgis*.*com/en/arcgis-online/administer/manage-licenses*.*htm*.

Before the start of interventions, the largest part of the country was meso- or hyperendemic for onchocerciasis, with only the most southern part being hypo-endemic [15]. There are two major river basins: Ôti basin and Mono basin. The 14 minor basins are: Asukawkaw, Gban, Huo, Kéran, Mô, Kpaza, Volta blanc, Kara (Ôti Basin) and Amou, Yoto, Haho, Zio, Ogou, Anié (Mono basin). In 2002, eleven onchocerciasis-endemic districts in the river basin Ôti (covering Kéran, Kara, and Mô rivers) across three regions in Togo (Savanes, Kara, Centrale) were classified as SIZ (as well as parts of Benin and Ghana) (**Fig 1**).

### Data collation

We collated epidemiological, entomological and programmatic data from Togo since the start of the OCP in 1974 from multiple data sources. Epidemiological data sources included the historical OCP database (“EPICROSS”), data provided by the Expanded Special Project for Elimination of Neglected Tropical Diseases (ESPEN) [22], grey and scientific literature, data received from the Togolese Ministry of Health, and evaluation data from the SIZ programme received from the former director of the SIZ programme (Dr. L. Yaméogo) [16]. From these sources, we extracted community-level microfiladermia (mf) prevalence data measured by skin snip (age-sex standardised or crude), community microfilarial load (CMFL), and the prevalence of antibody against OV16 IgG4 antigens (Rapid Diagnostic Test [RDT] and enzyme-linked immunosorbent assay [ELISA]). The full database, including a list of variables, is included in **S1 Database** and **S2 Text**, respectively. Definitions of the epidemiological indicators as used in this study are given in **Section B in S1 Text**.

Information on the history of vector control (i.e. start and end year of vector, potential interruptions) and data on annual biting rate (ABR) and annual transmission potential (ATP) were obtained from OCP’s EPICROSS database (which includes, among others, entomological data collected by the OCP Vector Control Unit field staff) and former SIZ-director, with additional information coming from grey and scientific literature. The start and end year of vector control per river basin were verified with the Togolese Ministry of Health. The grey literature was scrutinised to obtain better understanding of the geographical coverage and quality of vector control. Definitions of the entomological indicators are provided in **Section B in S1 Text**.

Data on the history of ivermectin mass treatment were obtained from 1) the MDA database as shared with us by the Togolese Ministry of Health reporting MDA therapeutic coverage at the village-level (1991–2002) and district-level (≥2002); 2) OCP’s EPICROSS database holding village-level treatment data for the period 1987–2000; 3) the publicly available ESPEN database on MDA for onchocerciasis, that contains district-level treatment data from 2014 onwards [22]; 4) and treatment data from the SIZ programme received from the former director of the SIZ programme for the period 2002–2005 (courtesy of Dr. L. Yaméogo) [16]. Key indicators extracted at village- or district level are the population at risk, population requiring treatment, population treated, number of MDA rounds provided, number of MDA rounds with ≥65% coverage, and therapeutic coverage. The grey literature was scrutinised to obtain better information on criteria applied in selecting villages for mass treatment, geographical coverage of treatment over time, potential interruptions in treatment. In some cases, we have village-level therapeutic coverage data from EPICROSS which are not reported by the Ministry of Health and vice versa. Particularly for the region of Kara for the years 1988–1991 and 1996–1997, the EPICROSS database seems to have more data than the Ministry of Health. It is likely that the initial villages that were sporadically treated for rolling-out the programme (1988–1991) were not recorded by the Ministry of Health. We do not have any village-level therapeutic coverage data from the Ministry of Health from any of the regions in Togo for the year 1997, and for the years 1998–1999 we only have data from Kara. As EPICROSS did report villages from Kara to have received treatment in those years, it likely indicates that the database by the Ministry of Health on ivermectin therapeutic coverage is not complete for the years <2002, and that the EPICROSS data can be considered supplementary.

### Data cleaning and technical validation

The data management and cleaning were carried out by the lead scientist (NVSVM). Data from various sources were merged using R statistical computing software [23], adding variables to indicate whether the location was part of the SIZ programme, data source, and further comments. Data cleaning concentrated on completing missing location-identifying variables and harmonising the identifying variables across records (e.g. correcting minor spelling variations, update administrative unit information according to the most recent demarcation, identifying and removing duplicate records, and adding or checking crude infection prevalence. See **Section A in S1 Text**, for a more detailed description of activities undertaking. We recorded the amendments in an R syntax. In case there was any doubt about the naming of a community or geographical unit, no changes were made. Onchocerciasis experts of the national onchocerciasis control programme in Togo were asked to validate any doubts and inconsistencies in the records.

### Data analysis

We visualised the programmatic, entomological and epidemiological time series data in scatter plots to reveal trends in the data. We assessed trends in mf prevalence over time based on the crude prevalence rather than the age-sex standardised prevalences reported by OCP (standardised to the OCP standard population [3]), as standardisation was rarely done in post-OCP surveys. Any entomological or epidemiological data collected before the local initiation of vector and MDA are considered to be pre-control data. Epidemiological data collated during the first 2 years of vector control, but before the initiation of MDA, may still give a good reflection of the pre-control situation and are therefore also included in analyses pertaining to the pre-control epidemiological situation. **Table 1** presents the start years of vector control. We mapped the location of each blackfly collection point since the start of entomological surveys in Togo, using ATP as outcome measure (**Fig C. in S1 Text**).

**Table 1:** Start and end year of vector control efforts in the major river basins and its tributaries in Togo.

Major river basin	Tributary	Vector control	
		Start year	End year
Asukawkaw	Wawa	1989	2002
	Yegué	1989	2002
Gban Houo	Domi	1988	2002
Haho	Haho	1988	2002
	Yoto	1988	2002
Kéran	Kéran (Ôti-Pendjari)	1977	2007
Kpaza	Agodeka	1978	1980/1997
Mô	Kama	1977	2007
	Katcha	1977	2007
	Mô (Ôti-Pendjari)	1977	2007
Mono	Anié	1989	2002
	Amou	1989	2002
	Kra	1989	2002
	Mono (Yambakopé, Bodowda, Kpime-Seva)	1989	2002
	Ogou (Oniakopé, Adibo)	1989	2002
Ôti	Bamoina	1977	2007
	Biankouri	1977	2007
	Kara (Koua, Poutouan)	1977	2007
	Koumongou	1977	2007
	Ôti-Pendjari (Sansougou)	1977	2007
Todzie	Todje	No VC	No VC

## Results

### Vector control

In Togo, vector control started in 1977 in the northern territories with its river basins of Ôti, Koumoungou and Kara. The central and southern regions were included into the vector control programme in 1988/1989 as part of OCP’s southern extension. The start year of vector control in Togo therefore varies between river basins, with the earliest river basins starting in 1977 and the latest in 1989 (**Table 1**). During some years of the early 1990’s, aerial larvicide application was suspended in several river basins of Togo [14]. In most river basins, vector control stopped with the closure of OCP in 2002. However, vector control continued for another five years under the SIZ programme in the Kéran, Mô and Ôti river basins, covering 11 districts across the regions of Savanes, Centrale, and Kara. In general, most of the onchocerciasis-endemic river basins in Togo implemented vector control activities for 14 years (e.g. Asukawkaw) to 25 years (e.g. Ôti).

### Mass drug administration with ivermectin

In 1988, MDA was introduced as a supplement to vector control in some areas of Togo. In most endemic districts, MDA was implemented annually from 1991 onwards. During the initial phases of OCP, only first-line villages (where the risk of eye or skin disease was highest, see **section B in S1 Text** (“First- and second-line villages”) were eligible for active ivermectin distribution through a vertical approach using mobile clinics, while treatment was only provided to individuals with clinical signs suggesting *O*. *volvulus* infection in settings with lower endemicity [24,25]. At that time, the reported therapeutic and geographic coverage was not very satisfactory [2]. There is a clear increase in reported villages that received treatment, according to the Ministry of Health database; from 47 villages reporting treatment in 1991 (range 1 to 8 villages per district, geographical coverage of 0.9%), to 245 villages in 1992 (range 2–59 villages per district, geographical coverage of 4.7%), to 1,019 villages in 1993 (range 2–250 villages per district, geographical coverage of 19.4%), to 3,867 villages in 2000 (range 1–45 villages per district, geographical coverage of 73.7%), to 5,226 villages in 2018 (range unknown, village-level data lacking, geographical coverage of 99.6%). By the end of OCP (2001–2002), the geographic coverage within districts ranged between 23% and 100% [26–29].

From 1997/1998 onwards, ivermectin was distributed through community-directed treatment with ivermectin, which increased both the achieved therapeutic and geographic coverage among MDA-eligible villages (first-line villages). Between 2000 and 2001, Togo conducted multiple onchocerciasis control activities such as community sensitisation, epidemiological and entomological surveillance, community-directed treatment with ivermectin, and training/re-training to detect and include villages, farms, and hamlets that required ivermectin treatment, enlarging the number of MDA-eligible villages [30] (MDA in first- and second-line villages).

The Ministry of Health in Togo reported that larger villages (≥2,000 population) were not treated with ivermectin until 2020. In all districts designated as SIZ, semi-annual MDA was introduced by 2003, and from 2014 onwards four more districts in the region of Plateaux received semi-annual MDA. A map of districts receiving annual or semi-annual MDA is presented in **Fig B in S1 Text**.

**Fig 2:**
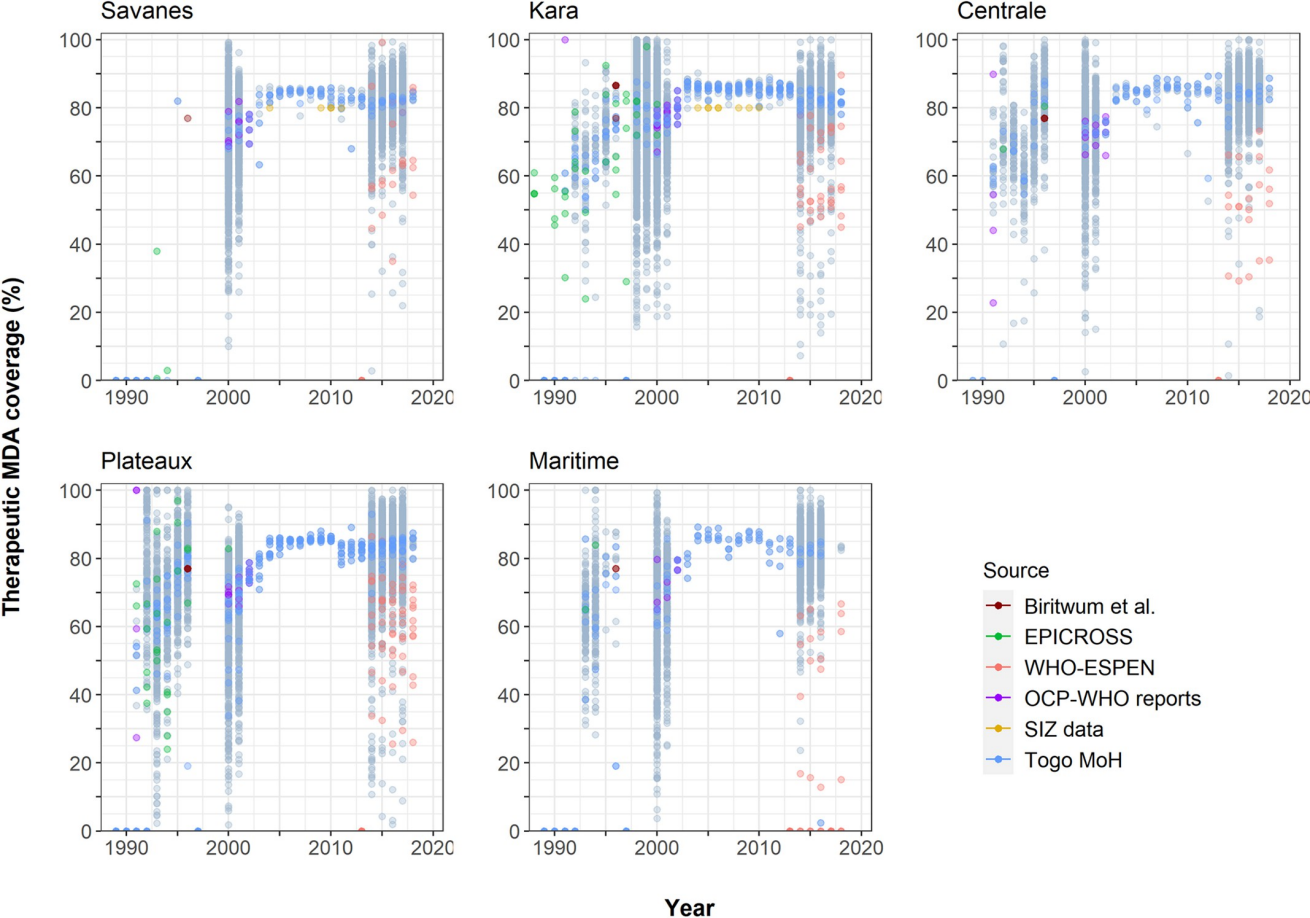
Reported village-level and district-level therapeutic coverage (%) of ivermectin mass drug administration per region in Togo over time. The grey dots represent the therapeutic coverage (in percentage) for each observed village (data obtained from the Togolese Ministry of Health). The coloured dots represent reported district-level therapeutic coverage per annum. The blue dots are data as provided by the Togolese Ministry of Health; the orange-red dots are data as provided by WHO-ESPEN portal; the purple dots are data as provided by JCP-OCP reports; the brown dots by Biritwum et al. [52]; and the green dots are data as provided by EPICROSS. Savanes and Kara, and areas of the Centrale region were considered to be Special Intervention Zones.

Village-level data on the therapeutic coverage are available from 1991–2001 and from 2017 onward, whereas only district-level therapeutic coverage data are available from 2002–2017. There is strong heterogeneity between villages in the reported therapeutic coverages for each year (light grey dots, **Fig 2**). The average village-level and district-level therapeutic coverage in Togo increased since the start of the MDA programme, until an average ~85% coverage was reached from 2002 onwards according to data from the Togolese Ministry of Health (**Fig 2**). However, there is quite some variability between data sources in the reported annual district-level mean therapeutic coverage over time (colours in **Fig 2**), related to the use of different definitions (see **Section B in S1 Text**). So while the coverage in villages targeted for treatment might be good, the district-level coverage appears poor in ESPEN data because the denominator includes population in villages not targeted for treatment. In several districts, we also see a drop in district-level mean therapeutic coverage for certain years [22], not necessarily implying that no ivermectin MDA was provided but potentially also related to the lack of quality data.

### Entomological data

**Fig 3:**
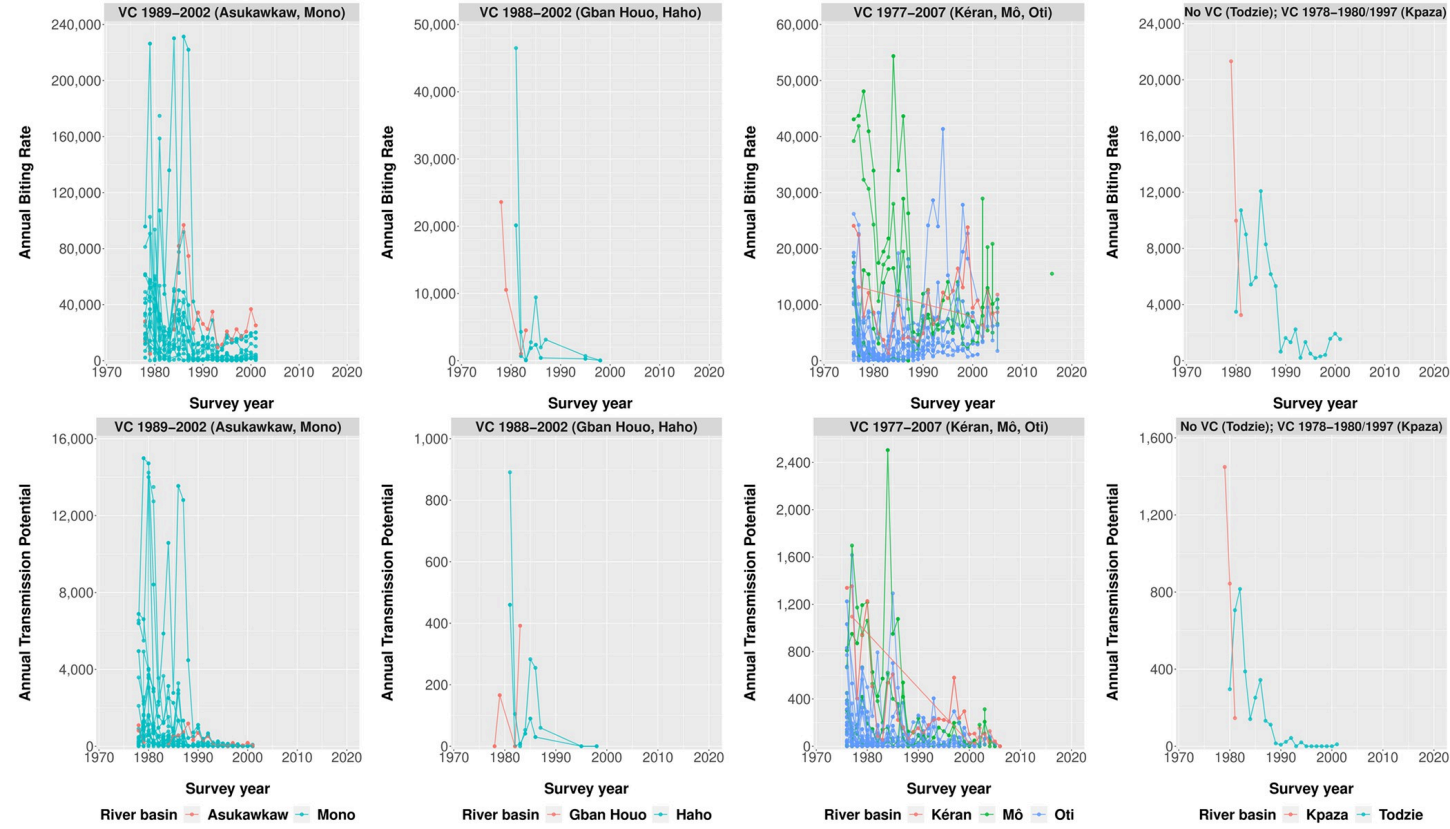
Time trends in the Annual Biting Rate (ABR) and Annual Transmission Potential (ATP) by major river basin, since the initiation of Onchocerciasis Control Programme in 1974 in Togo. The ABR is presented in the upper panels, and the ATP in the lower panels. Each panel represents the river basins with identical start and end year of vector control (VC); the vector control period and corresponding river basins indicated in the heading of each panel. The different dots are the various collection points in the country, and the lines connect the same collection points over survey year. The colours represent the various major river basins in the country. The river basins of Kéran, Mô, Ôti (third column panels) were designated as the Special Intervention Zone (SIZ).

We collated 1,349 records from 74 unique blackfly collection points in Togo covering the period from 1976 through 2016. In 1976–1977, ABR and ATP were measured in 37 collection points across the river basins in Kara, Mô and Kéran, all in areas that were designated as SIZ. The observed ABR varied between 130 and 43,712 (mean 10,106), with the ATP varying between 0 and 1,697 (mean 334). Considerably higher levels (up to 20,000) were later found in the Mono river basin, where vector control only started in 1989, with the highest ABRs observed in the collection points of Djodji and Tetetou (Plateaux region). ABRs were generally lower throughout the period of vector control, although they can be highly variable and sometimes ABRs during vector control were higher than the pre-control measurement (**Fig 3**, upper panels). In most catching points, the ABR remained between 1,030 and 20,852 since 1989 (exceptions of Asukawkaw river with an ABR of 36,795 in 2000 and Mô-Ôti river basin with an ABR of 28,934 in 2002). The ATP did drop nicely in all river basins. Assessments stopped in most areas in 2002, and were only continued in the designated SIZ areas where vector control continued through 2007. By then, ATP was low in all evaluated sites (**Fig 3**, lower panels). In 2016, ABR was measured once more in the collection point of Bouzalo (Mô-Ôti river basin, Centrale region) and estimated at 15,500. In 2015 some entomological surveys were performed in the Ôti river basin with over 5,500 blackflies examined by PCR across four collection points. No ABR was measured, but an overall crude *O*. *volvulus* infectivity rate in blackflies of 0.32% was reported.

### Epidemiological data

#### Overall

Our epidemiology database spans the period from 1975 through 2017 and it contains 1,864 surveys of mf prevalence for 478 unique villages and 755 records of OV16 IgG4 antibody prevalence from 625 unique villages (both RDT and ELISA). In 1988, epidemiological surveys began for sentinel villages of the OCP-Southern Extension in the river basins of Amou, Anie and Mono [14]. In total, over 366,000 people were skin snipped and 14,713 people were serologically tested using OV16 RDT or ELISA.

#### Skin snip results

The mean number of people skin skipped per village survey was 253 individuals with a range between 22 and 779 people examined. The database includes 136 pre-control records, with skin snip data from 74 unique communities, based on pre-control surveys performed between 1975 and 1989, depending on the local start year of interventions. The pre-control means crude mf prevalence varied between 7.8% and 89.4% (median 54.0%, interquartile range (IQR): 46.5%-66.7%). The pre-control standardised mf prevalence was somewhat higher (median 62.9%, IQR: 46.1%-67.0%). The pre-control median CMFL was 11.9 [IQR: 5.2–20.1]. The village Tchitchira had the highest pre-control crude mf prevalence (89.4% in 1976). This village is located close to the Kéran river in the Ôti river basin (Kéran district in Kara region).

**Fig 4:**
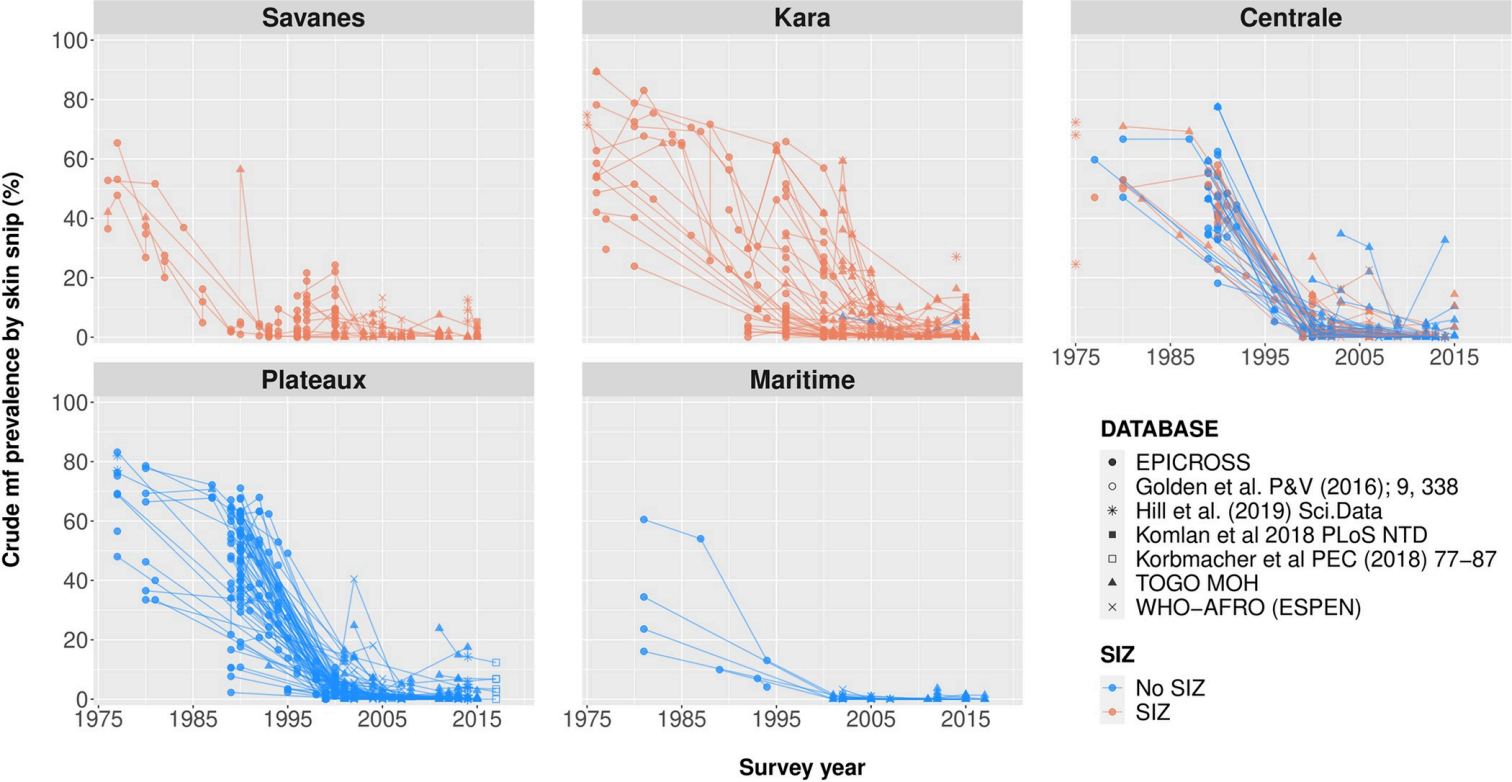
Crude mf prevalence over time by region, since the initiation of Onchocerciasis Control Programme in 1974 in Togo. The dots represent the reported crude mf prevalence per village, and the lines interconnect the same villages over various survey years. The various symbols represent the different data sources consulted, and the colours represent the location of the surveyed villages in Special Intervention Zone (SIZ, red) versus non-SIZ (blue) areas.

**Fig 5:**
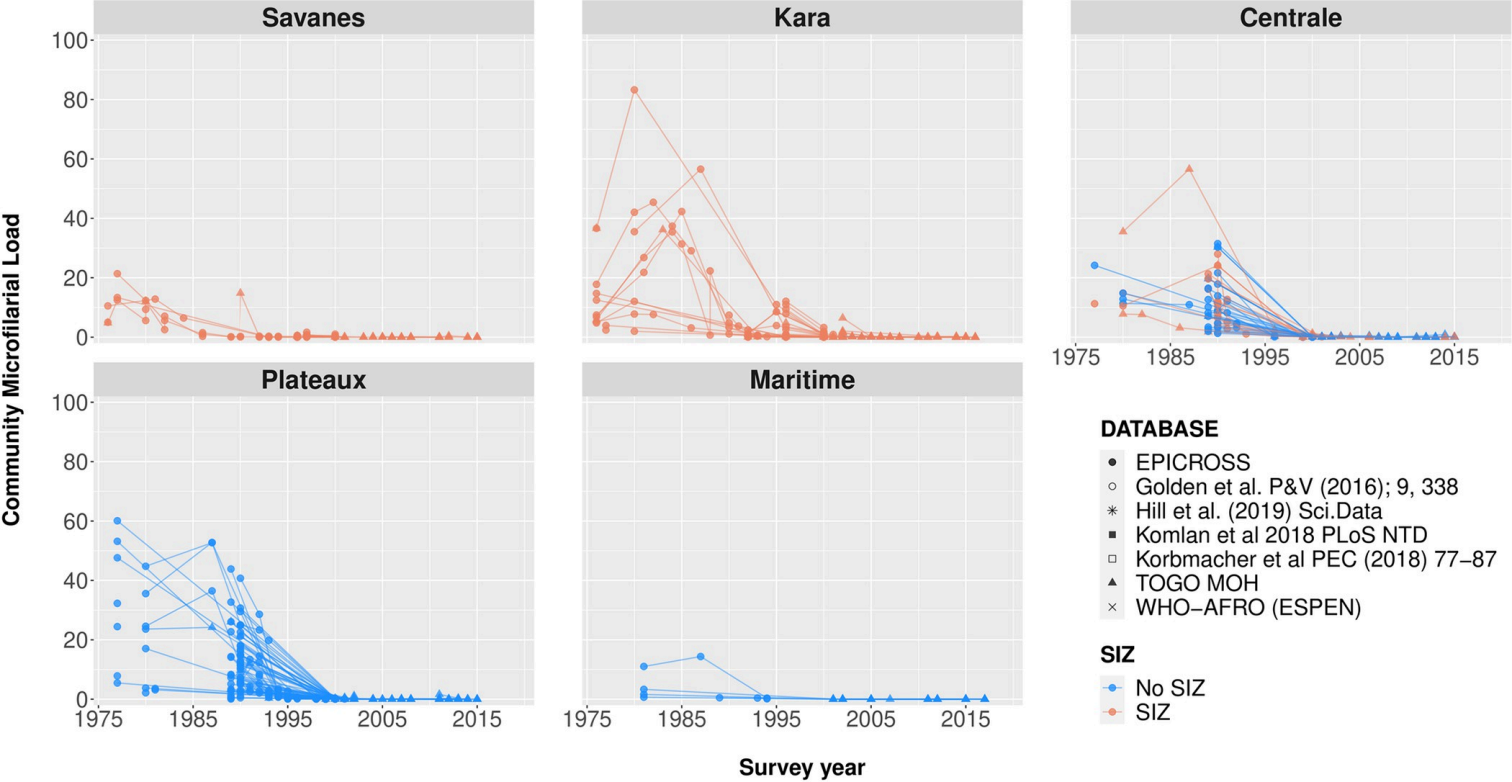
Community Microfilarial Load (CMFL) over time by region, since the initiation of Onchocerciasis Control Programme in 1974 in Togo. The dots represent the reported CMFL per village, and the lines interconnect the same villages over various survey years. The various symbols represent the different data sources consulted, and the colours represent the location of the surveyed villages in Special Intervention Zone (SIZ, red) versus non-SIZ (blue) areas. CMFL is defined as the geometric mean number of microfilariae per skin snip (mf/ss) among adults aged 20 years or more [3].

**Fig 4** shows the trend in crude mf prevalence by region. A strong decline in prevalence was seen since the start of interventions in most areas. Interventions seemed to have been least effective in the Kara region, where mf prevalence between 40% and 60% were still observed in the years 2000–2002. In the Centrale and Savanes regions, the interventions seemed more effective, although in the year 2000 there were still communities with mf prevalence above 20%. Closer examination of the data shows that high mf prevalences especially occurred around the Ôti Pendjari and the Ôti-tributaries Kéran, Kara and Mô (**Fig D-E** in **S1 Text**), i.e. in the regions Savanes and Kara and the districts Sotouboua in Centrale region (**Fig F-H** in **S1 Text**). This illustrates why the area was designated for special interventions. By 2014 and 2015, the mf prevalence had been reduced to very low levels in most districts, although there were still occasional villages with high observed mf prevalences ranging from 10.3% to 32.7% (6.4% of all surveys performed since 2014). Half of the recent mf prevalence surveys were performed in a research setting and the other half were performed by regular epidemiological surveys through the Ministry of Health. The three villages with highest mf prevalence are the villages of Batto (32.7%, Mô river basin, Centrale region), Wassite (26.0%, Kéran river basin, Kara region), Kpodji (17.5%, Mono river basin, Plateaux region). In some districts, mf prevalences seems to have increased slightly in the most recent years (**Fig F-J** in **S1 Text**). **Fig 5** shows that pre-control CMFL was particularly high in the regions of Kara, Centrale, and Plateaux, but dropped to (near) zero from the year 2000 onwards thanks to OCP control operations.

In former SIZ areas, 21.2% of all surveyed SIZ-villages (25/118) since 2012 still had a crude mf prevalence of ≥5%, and 9.3% reported a crude mf prevalence of ≥10% (11/118). Of the latter, the villages were located near the Kéran river basin (6/11), Oti (2/11), Mô (2/11), and Kpaza (1/11). These river basins cover the districts of Oti (Savanes region), Kéran and Bassar (Kara region), and Sotouboua (Centrale region).

#### OV16 results

Our database includes 751 records of the proportion of people with positive IgG4 responses to the recombinant *O*. *volvulus*-specific OV16 antigen, for all ages, people aged <20 years, or 5–9 year old children as tested by either OV16 ELISA or the commercial RDT. These data are from surveys performed in 2014–2017 in 619 unique communities; the data were obtained from the Togolese Ministry of Health and literature [18,19]. The mean number of people tested with OV16 per village survey was 20 (range 1–145), either by RDT or ELISA.

**Fig 6:**
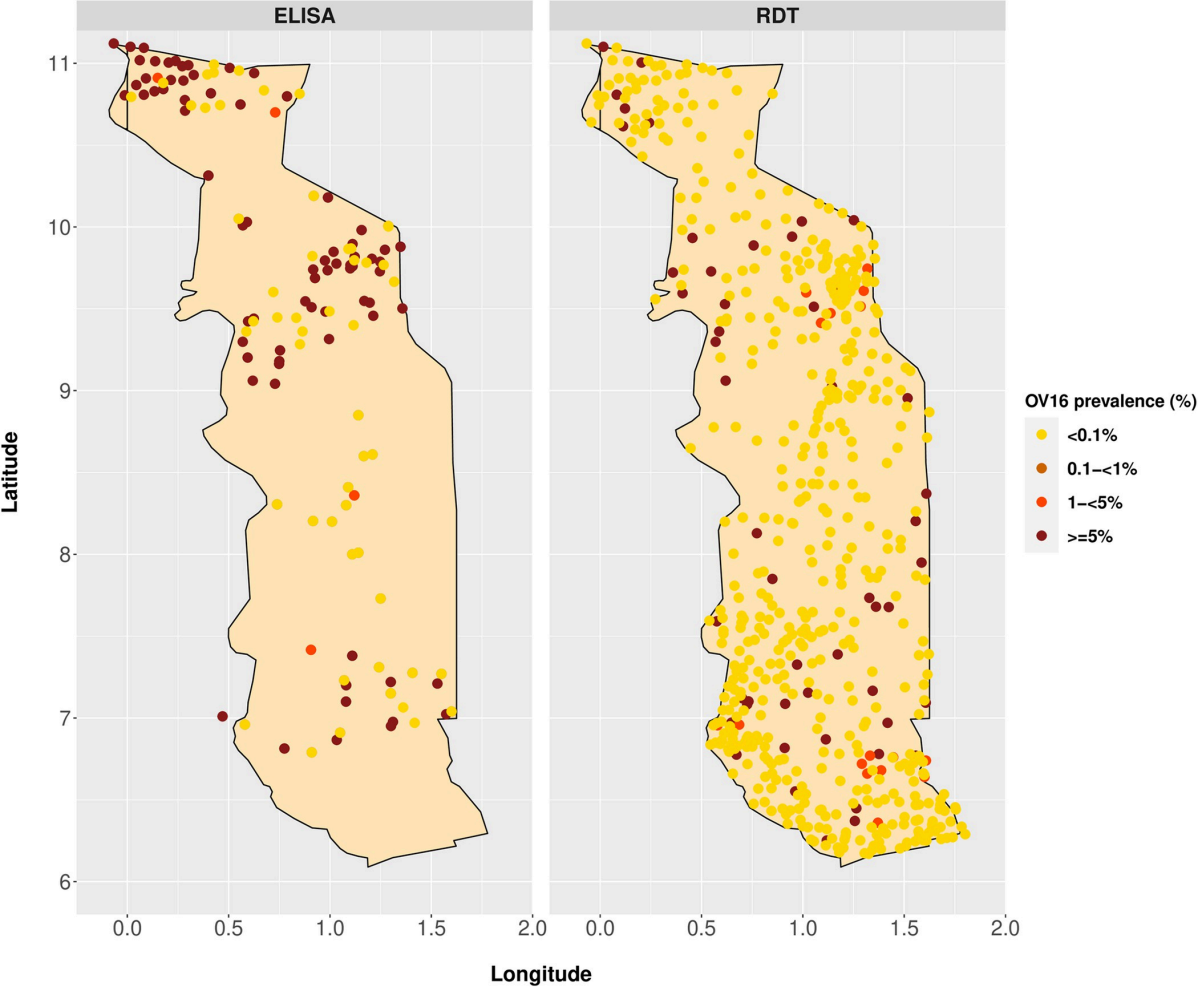
Map of the Republic of Togo with measured prevalence of anti-OV16 antibody positivity per community. Endemicity in OV16 prevalence was classified as <0.1% (as according to stop-MDA criteria), 0.1-<1% (low), 1-<5% (medium), ≥5% (high). The data points represent the various sample surveys where OV16 surveys were performed. The two panels present the different diagnostic tests used for OV16 prevalence measurements: ELISA (left) and commercial RDT (right). Data represent survey data collected between 2014–2017. Figure created in Rstudio [23]. We used the Rstudio packages cowplot, gridGraphics, ggmap, maps, and mapdata. The RStudio IDE is available under the GNU Affero General Public License v3 (Free Software).

**Fig 6** summarises the available data on OV16 IgG4 antibody prevalence. All-age OV16 surveys were performed in one to six villages per district. Particularly the results in the Kéran district of the Kara region show high variation in observed antibody prevalences between villages with a relatively high overall mean (>40%). On the other hand, all-age OV16 results using the commercial RDT in the districts of Kozah (Kara region) and Yoto (Maritime region) were very low, with seroprevalences between 0.0%-6.3% (sample size ranging between 47–145 across 21 villages) (data from Ministry of Health of Togo). The data from Kozah and Yoto districts were part of a study looking at different tests in a peri-elimination setting, and the sensitivity to infection was reported to be suboptimal. The OV16 IgG4 antibody prevalence among individuals below 20 years old was low in most districts, suggesting good progress to elimination (based on 34 surveys among this age group). The OV16 IgG4 antibody prevalence in this age group was still relatively high in Kéran district (Kara region) and Kpélé district. Four communities with individuals of <20 years of age were surveyed in Kéran district, reporting seroprevalences between 10.0% and 33.3% as determined by ELISA, although the number of people examined was quite low (ranging between 10–14 people). See also **Fig K-L** in **S1 Text** for further OV16 IgG4 antibody prevalence results by district and region.

There were 655 records of OV16 ELISA and RDT surveys performed among 6- to 9-year-old children across all five regions in Togo, sampled between 2015–2017. The mean OV16 seroprevalence of these surveys was 2.5% [range 0–66.7%]. Mean OV16 prevalence among this age group was 7.9% in the Savanes [range: 0–66.7%], 5.3% in the Kara [range: 0–54.0%], 1.3% in the Plateaux [range: 0–61.5%], 0.3% in the Maritime [range: 0–12.5%], and 0.2% in the Centrale region [range: 0–40.9%]. Generally, the mean OV16 prevalence when using the commercial RDT was 0.7% [range: 0–31.3%] and 13.3% [range: 0–66.7%] when using the ELISA (**Fig 6**). There are multiple villages where very high OV16 IgG4 antibody prevalences were reported among 5-to-9-year-old children, for example of 66.7% (Koutougou Solla, n = 4/6, 2015, Oti in Savanes), 60.0% (Tchore, n = 6/10, 2017, Tône in Savanes), and 61.5% (Leon, n = 8/13, 2017, Haho in Plateaux). Unfortunately, most of these results are based on very low number of children examined mean 15 children [range: 1–32]). Results of IgG4 or IgG1 responses to *O*. *volvulus* antigen (OvAg) are presented in **Section C in S1 Text**.

## Discussion

We presented a summary of data and visualised trends in infection and entomology over time, for the period between 1975 and 2017. We first discuss the data quality and completeness, and then move on to discuss the observed trends in infection indicators and the implications for the Togo national programme for onchocerciasis elimination.

### Data quality and completeness

There are some concerns about the quality and the completeness of the presented data. In the MDA database, it is likely that we do not have a complete overview of all villages treated with ivermectin before 2002 even though we combined multiple data sources. This may limit our understanding of the impact of MDA on onchocerciasis transmission by district or region over time.

In the entomology dataset, it was sometimes difficult to relate a catching point to a village, river basin, or district, and in some cases the geospatial coordinates were absent. The historical OCP database reports varying start and end years of vector control within river basins. It may be that vector control was continued longer in some parts of river basins. In addition, few entomological surveys were performed after the closure of OCP and SIZ and it is therefore not known how the cessation of vector control has affected trends in ATP and ABR. When the 11 OCP countries, including Togo, took over the responsibility of carrying out the residual activities of monitoring and the control of onchocerciasis after closure of OCP and/or SIZ, the countries faced several challenges including lack of technical assets, financial resources, or skilled personnel [31]. The lack of entomological data makes it difficult to assess current transmission conditions and intensity. Regular monitoring is required to track our efforts towards achieving onchocerciasis elimination. Results of an ABR of >1,000 and a declined ATP were reported in the Mô-Bouzalo and Mô-Kéméni river basins till 1999 using blackfly dissection, and in 2013–2014 by Johanns et al. [32]. In addition, Johanns et al. [32] suggest that ongoing transmission of *O*. *volvulus* occurs in the Mô river basin in central Togo in the years 2018 and 2019, with 0.58% and 0.45%, respectively, of *Simulium damnosum s*.*l*. vector blackflies carrying *O*. *volvulus* infections as determined by pooled real-time PCR.

In the epidemiological dataset, we encountered double records (same village, same survey year and month) with different reported crude mf prevalences, or villages without baseline epidemiological survey results (potentially related to changing district/region names over time). There have been some quality issues with the reported geographical coordinates of some of the villages, and sometimes various sources reported different coordinates for the same village. After we corrected the most obvious inaccuracies, data were shared again with the original data suppliers for further (opportunity to) data cleaning, validation, and authentication of the data, yet some inaccuracies in the data may have remained. An important limitation relating to the current status-quo of achieved progress—essential for policy-makers—is that most recent survey data are sometimes already old. Still, the compiled onchocerciasis database contains programmatic, entomological, and epidemiological (skin mf prevalence, OV16 prevalence, CMFL) data from Togo, and its overview will assist in understanding the historical onchocerciasis trends and current onchocerciasis status-quo in Togo, which can be useful for the preparation of elimination dossiers as requested by WHO [33].

The data used in this analysis are likely biased towards high prevalence communities. Because the initial goal of OCP was to eliminate onchocerciasis as public health and socio-economic problem, epidemiological assessments during OCP were more likely to be performed among villages with close proximity to rivers (breeding sites) or first villages near rivers with uninhabited ground between the village and river [34] where the risk of blinding onchocerciasis was believed to be highest. It may be that prevalence rates of endemic villages further away from breeding sites, or those close to man-made lakes that were not treated using insecticides (due to use of river water for drinking and fish industry [35]), are not included in our database due to the absence of epidemiological surveys from these villages. Scrutinization of old OCP reports might resolve some of these unknowns.

### Importance of contextual information

There are significant discrepancies in the reported therapeutic coverages across data sources, survey years and villages. Unfortunately, we only have village-level data for the period of 1988–2001 and till 2002 for Kara, and the period of 2014–2017 (till 2016 for Maritime). We have limited information on the total number of MDA-eligible villages per district, and henceforth we cannot calculate the (district-level) geographic coverage, or record any missed rounds in the past. The district-level therapeutic coverage was calculated based on the available village-level therapeutic coverages within the respective district, and there clearly is much heterogeneity between the villages and between the district- and village-level coverages. Particularly in Kara and Plateaux there are still some villages with very poor therapeutic coverage (2011–2018). More precise information of the MDA history for all MDA-eligible villages would be useful to better understand the presented trends in infection prevalence. Original treatment area boundaries and criteria for MDA eligibility (e.g. relating to distance to the river of first/second/third line villages, or population size), and any changes therein over time, are opaque.

The district-level therapeutic coverages as reported by ESPEN for MDA distributed between 2014–2018 are significantly lower than those reported by the Ministry of Health. There is an important caveat here that affects the appearance of the ESPEN data: ESPEN calculated the coverage using the total population of the entire district as denominator, whereas the onchocerciasis programme in Togo only targets some of the villages in each district. Therefore, ESPEN estimates are much lower than the Togolese programmatic data, due to the use of different denominators. ESPEN calculates the MDA coverage per district by dividing the number of people treated by the total population within a district, regardless of onchocerciasis endemicity (thus potentially including non-endemic villages). In contrast, the Ministry of Health of Togo calculates the MDA coverage by dividing the number of treated people through the total population in villages targeted for treatment (potentially missing endemic villages that are located outside the treatment area). This difference in use of population denominator is essential for understanding the programmatic data of Togo. This knowledge may also assist in understanding the performance of onchocerciasis programmes within other onchocerciasis-endemic countries.

### Observed trends in infection

Thanks to vector control and mass distribution of ivermectin, infection prevalence has declined strongly across the country. In the majority (313/358, 87.4%) of the villages across Togo surveyed since 2012, the overall crude *O*. *volvulus* mf prevalence was below 5%. However, in several locations along the river basins of Amou (Mono), Kara, Kéran, Ôti (Penjari), and Mô, the mf positivity still ranged between 5.0% and 32.7%, suggesting continued transmission. Since 2012, 21.2% of all surveyed villages formally under SIZ still had a crude mf prevalence of ≥5%, and 9.3% reported a crude mf prevalence of ≥10% (11/118). This shows that, among the limited number of villages surveyed since 2012 in former SIZ-areas, the epidemiological results are not yet satisfactory.

This study assists in identifying areas where current transmission likely still occurs or where a further situation analysis of onchocerciasis transmission should be performed. This enables more efficient use of limited financial and human resources. The epidemiological data suggests potential ongoing transmission in the districts of Oti and Kpendjal, with potential cross-district transmission to Tandjouaré (Savanes region), in the districts of Bassar and Kéran (Kara region), Satouboua (Centrale region), and in the districts of Amou and Haho (Plateaux). As these districts within Plateaux are not bordering, surrounding districts within the region should also be suspected of ongoing onchocerciasis transmission. The river basins of Kéran, Oti, and Mô indeed flow through the aforementioned districts within the Savanes, Kara and Centrale regions. It is recommended to perform more wide-scale surveys in these districts and to continue onchocerciasis control efforts until more evidence becomes available. Particularly these areas can then more rapidly be targeted for intensified interventions, which may accelerate the national onchocerciasis elimination efforts.

### OV16 data

Togo has taken initiative to monitor and evaluate (M&E) the overall programmatic impact on infection prevalence and to report country achievements. These country M&E survey data allow us to make inferences about the status of transmission/impact of treatment. The OV16 data are mostly consistent with the mf prevalence data. Although some districts have a mean <0.1% serology prevalence, there are still villages found with high OV16 IgG4 antibody prevalence within the same districts (up to 20.0%). Examples are within the districts of Kozah (Kara), Tchaoudjo and Blitta (Centrale region), Agou, Akébou, Amou, Kloto, Ogou, Wawa (Plateaux region), and Avé, Golfe, Zio (Maritime). These districts may be approaching successful reduction of onchocerciasis transmission, but caution must be made due to heterogeneity between villages in these districts. More effort is still needed in primarily the districts of Cinkassé, Oti and Tône (Savanes), Bassar, Dankpen, Doufelgou, and Kéran (Kara), Anié, Haho and Kpélé (Plateaux) due to high serology prevalences in multiple villages. Nonetheless very satisfactory OV16 results were reported among children <10 years, with eight districts that are on track towards onchocerciasis elimination (Assoli in Kara; Sotouboua and Tchamba in Centrale; Danyi in Plateaux; Bas-Mono, Lacs, Vo and Yoto in Maritime) with no infection found among the sampled children. These data show an impressive impact on transmission thanks to long-term interventions, although in most districts of Togo, ivermectin MDA should be continued until the next round of serological surveys among children <10 years of age.

The Ministry of Health in Togo performed another survey in 2017 (school-based) comparing OV16 RDT (on finger-stick whole blood in the field) to the OV16 ELISA (the HRP ELISA, which evolved into the SD ELISA kit). The study included a total of 2,654 children and showed that 0.5% of children were positive by RDT read at 20 min (the standard at that time), whereas 2.8% of children were positive by RDT read at 24 h, and 8.1% children were positive by ELISA (village-level data not available to us). The concordance between RDT and ELISA was reported to be poor [36]. In districts where villages were found with high recent mf or OV16 IgG4 antibody prevalences, more serological evaluations should be performed targeting children in the age of 5–9 years preferably combined with entomological assessments to understand the current transmission status. Also, it would be better to harmonise epidemiological assessment protocols and follow the WHO recommendations in this area.

There are some other caveats in the interpretation of the OV16 data, namely different target populations (sampling or population-wide assessments in high-risk or historically high-transmission villages versus near-elimination villages), variable age-composition of population samples for which we could not adjust, and different sampling strategies (at random versus purposive selection of first-line, second-line, or third-line villages). Different settings where OV16 surveys are performed also matter in the interpretation of the results. For example, the districts of Kozah and Yoto were selected as part of a study looking at different tests in a near-elimination setting. Contrarily, the Korbmacher [37] and Komlan [18] studies were specifically looking for places where transmission might be ongoing (surveys performed in former SIZ areas). It is important to make the distinction between these studies because in each instance the investigators demonstrated what they thought they might find (higher transmission where they thought transmission might be ongoing, lower transmission where they thought it was low or potentially interrupted). Still, observations of zero OV16 IgG4 antibody prevalence suggests that elimination may be near, whereas relatively high prevalences are suggestive of ongoing transmission. Also in areas where elimination is suspected, serological surveys should be done, specifically targeting 5-to-9-year-old children to align with performance targets in onchocerciasis elimination efforts (target of 0.1% OV16 IgG4 antibody prevalence in children aged 5–9 years [38]).

### Why elimination has not yet been achieved

Even though infection prevalence declined substantially in Togo and elimination may be suspected in some areas, more efforts are required to achieve country-wide elimination. Many high endemicity areas became medium or low endemic thanks to long-term interventions. Various factors may explain why elimination is not yet achieved despite decades-long interventions.

Firstly, while vector control had been highly successful in the core area of OCP, flies continued to appear in the peripheral areas, including the northern part of Togo, due to reinvasion of flies from more southern/eastern located river basins that were not included in the treatment [39]. In some years, exceptional hydrological conditions may also have contributed to reinvasion [40]. The geography of certain river basins in Togo also contributes to local persistent *O*. *volvulus* transmission. The river networks in northern and central Togo are extensive with cross-river and cross-border flows of the Kéran river (flowing in the Koumoungou and Ôti), Ôti (flowing from Benin crossing Togo from east to west), and Mô (flowing from Togo into the Volta river basin in Ghana. These river conditions allow for trans-border migration of vectors over various river basins [41]. The savanna *S*. *damnosum s*.*l*. species dominates the northern-central regions of Togo. These species are known to migrate large distances during the rainy seasons [42,43]. Even during the dry season, transmission of onchocerciasis by *S*. *squamosum* at the Amou-Oblo (Mono) river basin has been reported with high biting and infectivity rates [44]. This makes many river basins of Togo favourable for persistence of blackflies and *O*. *volvulus* infectivity. The geographical characteristics of the river basins stretching into Benin and Ghana support the expansion of control efforts towards cross-border collaborations [45]. Overall, epidemiological achievements were lower than expected, as OCP reported in its progress report [46].

Secondly, for several reasons the impact of MDA throughout the years may also have been suboptimal, according to current treatment guidelines and considering current elimination targets. When MDA was first implemented in Togo, it was specifically targeted at high endemic villages (CMFL >5 mf/s, according to [47]). The effectiveness of MDA may have been compromised because a part of the parasite population remained untreated with ivermectin (e.g., medium or low endemic villages). Also, initially MDA was provided by national teams, which has been challenging and may have resulted in low coverages. Between the years 1999–2002, the Ministry of Health data showed a drop in mean therapeutic coverage. Personal communication with the National Onchocerciasis Programme mentioned challenges with the delivery of mass treatment as funding would run out due to unexpected obstacles during the skin snip assessments prior to MDA. This has possibly resulted in some villages going untreated (villages with 0% coverages are not reported in our MDA database, thus not visualised). Lastly, geographical coverage of ivermectin mass treatment may have been further compromised by the practice to treat only part of large communities with >2,000 individuals or not treat them at all. This may have led to an overestimation of the geographical coverage as the numerator has likely been underestimated.

Thirdly, human migration (e.g. temporary seasonal work) may also have contributed to suboptimal coverages and influx of infection from surrounding areas. One study found that particularly males aged 15 to 40 years were absent during skin examinations and treatment rounds due to seasonal migration [18]. Again, (continued) cross-border collaboration in the near future should address this issue.

Finally, there are some challenges in current adherence to ivermectin intake. Multiple factors could contribute to low adherence, including programme fatigue, fear of side-effects, absence during MDA, pregnancy, the desire to drink alcohol, poor opinion about the MDA campaign, or drug distribution challenges [48,49]. Another reason is that the new generation of people have never experienced the presence of river blindness in the communities, and thus have lower awareness of the disease including MDA participation [50]. Mitigation or control measures associated with the emergence of new diseases, such as during the SARS-CoV-2 pandemic, could affect the quality and continuity of MDA in the context of onchocerciasis and other NTDs.

### Concept for data scrutinisation and visualisation

The onchocerciasis control programme of Togo has collected vast amounts of infection, entomological and programmatic data. About 46 years of data is safeguarded at the country-level, but to monitor and evaluate progress towards elimination and make programmatic decisions, it is imperative to compile and review the data. A method for data scrutinisation and visualisation was used to understand history of control (vector control and MDA), entomological progress (ABR and ATP) and trends in infection (mf skin snip, OV16, and where available, OvAg). Here we have described the process by which other onchocerciasis programmes could pull their data together and begin to examine it in order to understand the impact of their work and perhaps identify next steps for programmatic decision making.

This works suggests that there is likely limited remaining ongoing transmission in the districts of Assoli, Avé, Binah, Kozah, Tandjouaré, Tône, and Zio. Possibility of scaling down MDA may be considered in these areas. Other districts, i.e., Kpendjal, Ôti, Kéran, Bassar, Sotouboua, Amou and Haho possibly still have eminent ongoing transmission, and likely require continued and intensified control efforts. In general, more conformation mapping and surveys are required to assess the current progress towards elimination in Togo.

## Conclusion

We show that vector control and annual or more frequent ivermectin treatment in Togo resulted in a strong decline in onchocerciasis prevalence to low levels in most villages. Unfortunately, high prevalence of skin mf (≥5%) persists in more than 11% of surveyed villages and in other villages high OV16 prevalence has been demonstrated in children in 2014 and 2017. These results provide evidence of ongoing transmission in the river basins of Amou (Mono), Kara, Kéran, Ôti (Penjari), and Mô, which could be explained by the climate and the geographical conditions of the river networks in Togo. Targeting onchocerciasis elimination in large parts of Togo by 2030 therefore calls for continuous, intensified, and well-adapted interventions aiming at optimal geographic and therapeutic coverage of ivermectin MDA, targeting pockets of high transmission or high-risk populations. Continued and renewed entomological and epidemiological monitoring for onchocerciasis should be performed, particularly among villages in the aforementioned at-risk districts. These efforts should be harmonised with neighbouring countries to attain the elimination goals as outlined in the WHO Road Map for NTDs 2021–2030 [8,51]. We suggest similar exercises for other former OCP-countries to scrutinise and visualise their available data since the start of the OCP programme, in order to make important programmatic decisions.

## Supporting information

S1 Database Available databases; 1) skin snip; 2) OV16; 3) Other; 4) Entomology; 5) MDA history village-level; 6) MDA history district level. (XLSX)

S1 Text Detailed methods and results. (PDF)

S2 Text Explanation of variables of the databases. (PDF)
