# Supplementary material for: Impact of ivermectin and vector control on onchocerciasis transmission in Togo: Assessing the empirical evidence on trends in infection and entomological indicators
Source: PLoS Negl Trop Dis. 2024 Jul 22;18(7):e0012312. doi: 10.1371/journal.pntd.0012312 (PMC11293710; doi:10.1371/journal.pntd.0012312)
Supplement: S1 Text — (PDF) [file pntd.0012312.s002.pdf]

S1 Text: Detailed methods and results

Supplement to:

Impact of ivermectin and vector control on onchocerciasis transmission in Togo: assessing the empirical evidence on trends in infection and entomological indicators.

Authors:

Natalie V.S. Vinkeles Melchers[1,2#], Sibabi Agoro[3], Kwamy Togbey[3], Koffi Padjoudoum[3], Ibrahim Gado Telou[3], Potchoziou Karabou[3], Touka Djatho[3], Michel Datagni[3], Ameyo Monique Dorkenoo[4], Yao Kassankogno[5]†, Rachel Bronzan[6], Wilma A. Stolk[2]

Author affiliation:

1. Health & Society Group, Social Sciences Department, Wageningen University & Research, Wageningen, The Netherlands.
2. Department of Public Health, Erasmus MC, University Medical Center Rotterdam, Rotterdam, The Netherlands.
3. National Institute of Hygiene, Ministry of Health, Public Hygiene and Universal Access to Care, Lomé, Togo.
4. Faculté Des Sciences de La Santé, Université de Lomé, Boulevard Eyadema, 01BP 1515, Lomé, Togo.
5. Health and Development International (HDI), Lomé, Togo.
6. Bill & Melinda Gates Foundation, 500 5th Ave N, Seattle, WA 98109, United States of America.

Correspondence to:

Natalie V.S. Vinkeles Melchers, PhD. MSc. MPH.

Health & Society Group, Social Sciences Department, Wageningen University & Research, Hollandseweg 1, 6706 KN Wageningen, The Netherlands.

Email: natalie.vinkelesmelchers@wur.nl

† Deceased.

Table of contents

S1 Text: Detailed methods and results 0
Table of contents 1
Section 1. Geographic identification information 2
Region, districts and river systems 2
Geographic identification variables 3
Completing, updating and correcting geographic identification information 3
Section 2. Epidemiological and entomological definitions 5
Crude microfilariae (mf) prevalence 5
Standardised microfilariae (mf) prevalence 5
OV16 seroprevalence 5
Community microfilarial load 5
First- and second-line villages 5
Therapeutic coverage of ivermectin treatment 6
Geographic coverage of ivermectin distribution 6
Annual Biting Rate 6
Annual Transmission Potential 7
Section 3. Additional results and figures 7
Mass Drug Administration 7
Entomology 8
Epidemiology 10
Additional OV16 results 15
Results of *O. volvulus* antigen tests (OvAg) 17
References 18

Section A. Geographic identification information

Region, districts and river systems

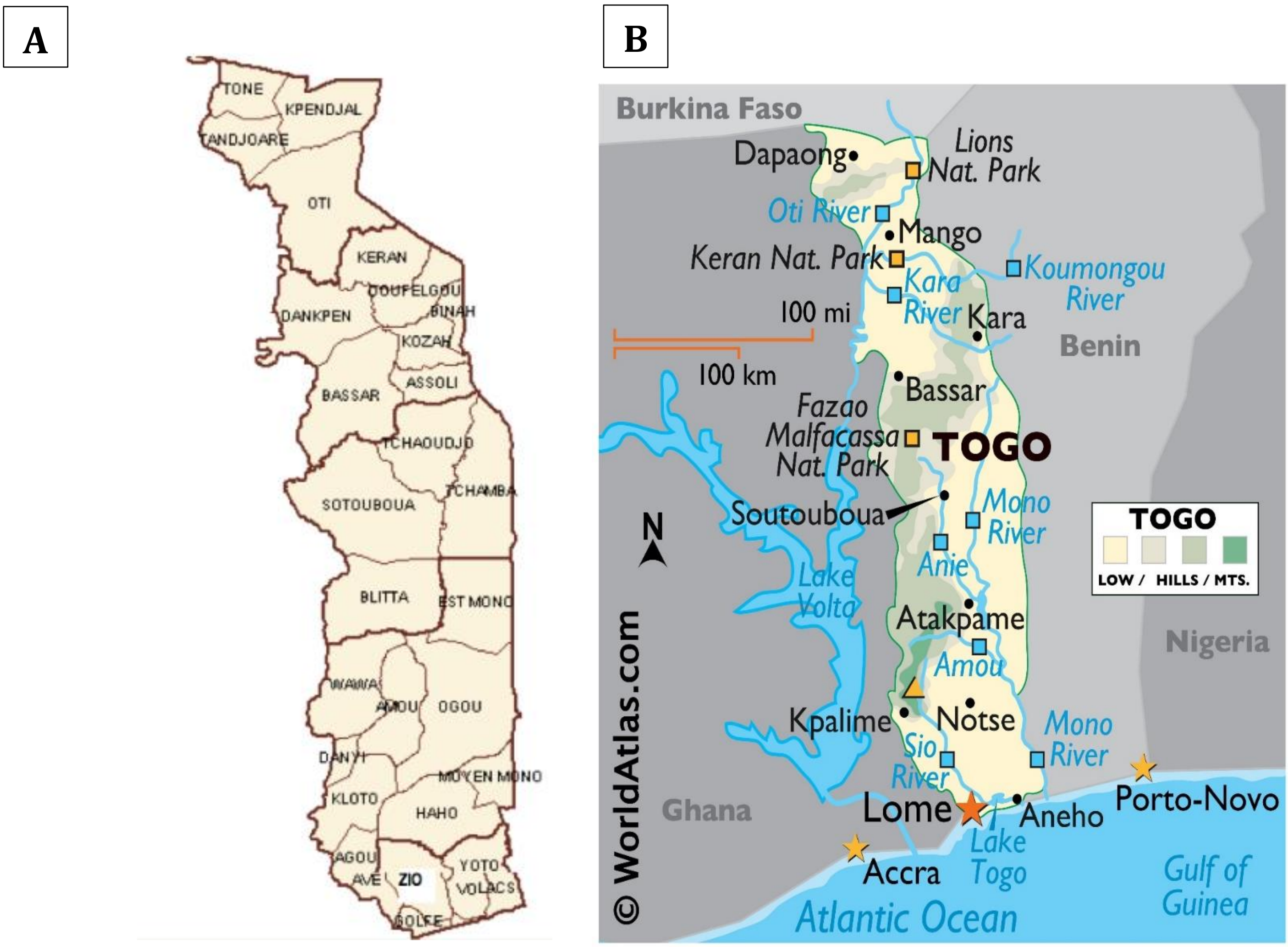

Fig A. Map of the Togo with A) its regions and districts (prefectures, second level administrative divisions) by 2018; and B) major river basins.
Figure A;A) with the courtesy of Rachel Bronzan, created in ArcGIS (https://www.esri.com/en-us/arcgis/products/arcgis-desktop/resources).
Base layers of the map: https://data.humdata.org/dataset/cod-ab-tgo.
Shape files are in " tgo_adm_inseed_itos_20210107_SHP.zip".

Geographic identification variables

We include the following geographic identification variables in our databases:

- Village name
- Country name
- Name of administrative division (first and second level)
- Geopositioning data (latitude and longitude)
- Name of river basin and grand river basin (only in epidemiology and entomology database)
- Closest fly collection point used by OCP
- Name of collection point
- Whether or not the location was part of the special intervention zone from 2002 onward (manually added)
- OCP phase name
- IU name (only in MDA database)

Completing, updating and correcting geographic identification information

1. To prevent errors when merging data from different sources, all names (country, village, prefecture, region, river basin, etc) were capitalised and leading and trailing blanks were removed. Other inconsistencies in spelling of names were corrected manually when detected during the merging of multiple databases. In case there was any doubt about the naming of a community or geographical unit, no changes were made.
2. Names of regions and districts (prefectures) were used according to the administrative divisions by 2018 (**Fig A**). We made some minor corrections in naming of districts to adapt to the administrative levels by 2018. We cross-checked villages for which multiple epidemiological surveys were carried out, and, if necessary, we replaced the former district name by the new district name of the respective villages.
3. Geographical coordinates were checked, corrected or completely as opportune.
    a. If village names were the only identifiable variable, and the same village was already used in EPICROSS or the ESPEN database for a different survey year and that record contained more information on river basis, districts, or regions, the location specifics were copied.
    b. If missing geographical coordinates could not be completed from other sources, or if given geographical coordinates were obviously erroneous (e.g. because the coordinates fall in another country) or inconsistent across data sources, we manually searched for the correct geographical coordinates of the location in the online GIS database (www.diva-gis.org) or on Google Maps and made the

necessary corrections. If correct geographical coordinates could not be identified in that way, the coordinates were reported as not available (NA). We visually verified the correctness of geospatial coordinates by mapping the locations onto a country map, using the statistical computing programme R (packages "ggmap", "maps", "mapdata").

4. Potential duplicate data records were investigated and removed if necessary. We removed double entries when the following variables per record were identical: country, location name, river basin, survey year, number of people examined and positive, crude prevalence, diagnostic method.
5. Where possible, we used the total population examined and total population positive within a village and per survey year to calculate (anew) the crude mf prevalence.

Section B. Epidemiological and entomological definitions

Crude microfilariae (mf) prevalence

Number of individuals with positive skin snip over the total number of people examined during the time of an epidemiological survey. In case multiple snips were taken, e.g. from the left and right iliac crest, at least one of them should be positive.

Standardised microfilariae (mf) prevalence

Age-sex adjusted mf prevalences, reflecting the mf prevalence that would have been found in a population with the age-sex structure of the OCP reference population [1]. It was standard practice in OCP to report standardised mf prevalences.

OV16 seroprevalence

Percentage of individuals with an IgG4 antibody response against the recombinant *Onchocerca volvulus*, calculated as the number of seropositives over the total population examined during the time of an epidemiological survey. An Ov16 prevalence of <0.1% in children under 10 years of age is defined as the critical threshold for interruption or elimination of transmission [2].

Community microfilarial load

The community microfilarial load (CMFL) is calculated as the geometric mean of the number of microfilariae per skin snip (mf/ss) in adults aged 20 years and older [3]. To avoid problems with zero mf counts, the mean is calculated after a log(n + 1) transformation, where n = the number of mf/ss. The CMFL is an indicator of the intensity of infection in a community.

First- and second-line villages

A first-line village is the closest village to a blackfly breeding site; a second-line village is the second closest villages to that same blackfly breeding site. The first- and second-line villages were identified through a historical risk assessment survey of multiple villages from which a list of all MDA-eligible villages was developed. Historically, at random third-line villages were identified that were located further away from potential breeding sites. In more recent years, the national onchocerciasis programme of Togo considered villages that were not first-line or second-line to be third-line villages. The major determinant for the definition of a first-line village was henceforth the location of the village as compared to the nearest blackfly breeding site. A secondary determinant for the definition of a first-line village was also whether the zone between the village and the breeding site contains other settlements. If the zone in between is uninhabited, the village is called first-line with maximum contacts with the biting blackflies coming from the rivers. The second- and third-line villages were assumed to profit from a natural barrier or buffer

provided by the first-line village. Some villages may thus be defined as first-line villages, although the villages may have located at a fair distance from the rivers [3,4].

Therapeutic coverage of ivermectin treatment

Percentage of population that is treated with ivermectin in total census population. The total census population includes those who are ineligible for treatment (i.e., children under 5 years old, people with contra-indications (e.g. certain illnesses, pregnant women). The Togolese Ministry of Health calculated the therapeutic coverage at village-level as the total number of people within a village that received ivermectin divided by the total number of people present at the time of a MDA campaign of an MDA-eligible village (affecting the MoH database). MDA-eligible villages as defined by the Togolese Ministry of Health constituted of villages at high risk of onchocerciasis and of a population size of <2,000 people, since the start of community-directed treatment with ivermectin (CDTI) till 2020. The district-level therapeutic coverage is calculated as the mean coverage of MDA that has been reached among eligible villages. This definition is applied by most data sources. ESPEN, however, reports district-level therapeutic coverage as the number of people treated with ivermectin divided by the entire population-at-risk of an endemic district [5]. ESPEN thus considers the entire district at-risk if there is treatment going on in the district, so every village is included in the denominator whereas only some villages are actually targeted for treatment.

Geographic coverage of ivermectin distribution

Percentage of communities that received treatment out of the total eligible (endemic) communities in the area of interest.

Annual Biting Rate

The annual biting rate (ABR) is the estimated number of *Simulium* bites that a person exposed to a *Simulium* vector population will receive in a year. The ABR is calculated as follows, from human landing catch data [6]:

$$ABR = \frac{No.of\ flies\ caught\ \times\ No.of\ days\ in\ year}{No.of\ catching\ days}$$

The ABR is affected by vector control in a given area, but presumably not by ivermectin treatment. The ABR is an indicator of human exposure to *Simulium* fly bites [7]. During the OCP, the WHO considered entomological results as satisfactory when the ABR across savanna areas in West-Africa was less than 1,000 bites/person/year [8].

Annual Transmission Potential

The crude annual transmission potential (ATP) is the estimated number of *O. volvulus* L3 larvae which would have been transmitted to a person if exposed to a *Simulium* vector population during a year [6]. This version of the ATP formula was used when flies were dissected (rather than PCR screening, which is available these days). L3 larvae can be isolated from blackflies by dissection using a dissecting microscope and the developmental stages of larvae can be scored (L1-L3) [9]. L3 stages were than found in the head, thorax, and abdomen. The crude ATP included all *Onchocerca* species as *Onchocerca* sub-species cannot be morphologically distinguished by light microscopy [7]. The ATP was calculated as follows [6]:

$$ATP = \frac{ABR \times No.of\ all\ L3\ larvae}{No.of\ flies\ dissected\ annually}$$

The ATP is an indicator of human exposure to infectious *Simulium* bites, and therefore directly measures ongoing transmission. During the OCP, the WHO considered entomological results as satisfactory when the ATP was less than 100 L3 larvae/per son/year across savanna areas in West-Africa [8]. More recently, the WHO (2016) stated that an annual rate of 20 L3 larvae/person/year is a suitable cut-off point for onchocerciasis elimination [2].

Section C. Additional results and figures

Mass Drug Administration

Fig B shows a map of districts in Togo that receive either annual or semi-annual MDA from 2014 onwards. Out of the 40 districts in Togo, 32 are MDA-eligible and receive treatment, out of which 15 receive semi-annual MDA.

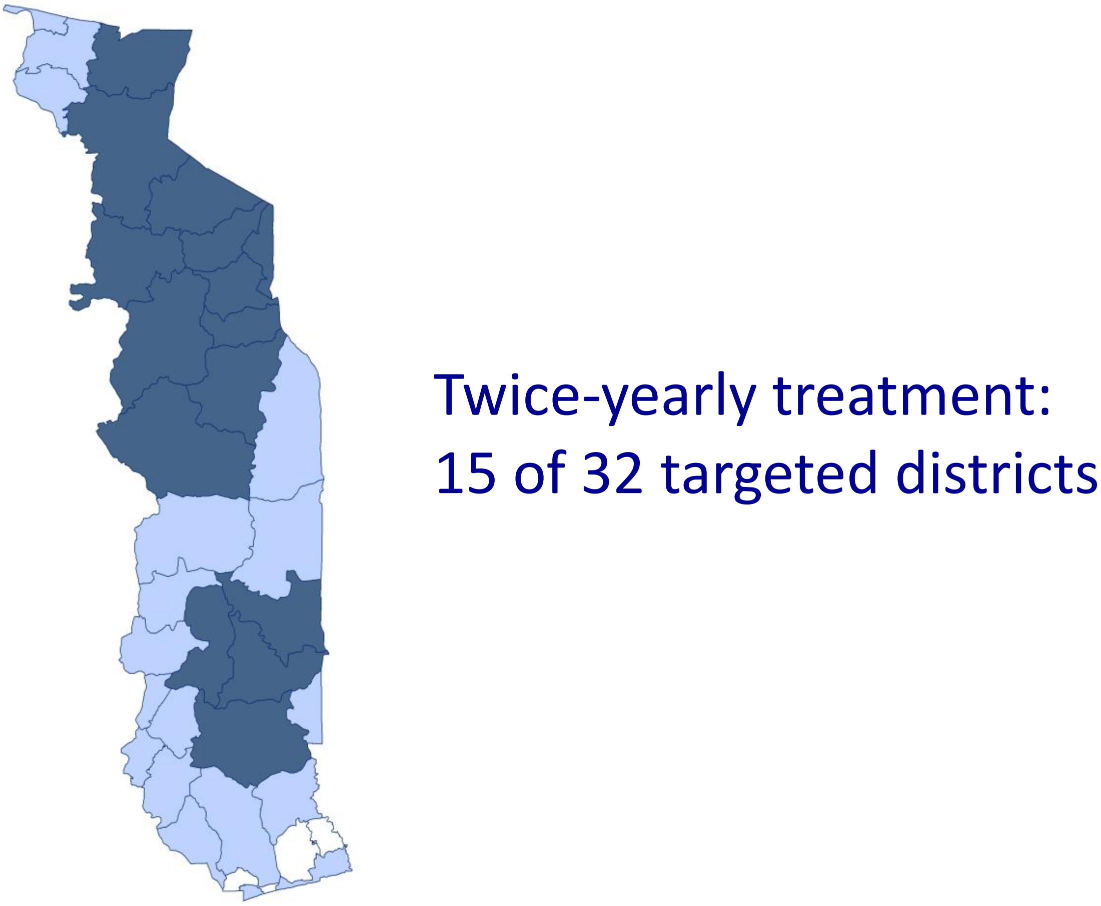

Fig B. A map of districts in Togo that receive either annual or semi-annual MDA from 2014 onwards. Out of the 32 MDA-eligible districts, 15 districts received semi-annual MDA (dark blue) and 17 districts received annual MDA (light blue). The districts in white are considered to be non-endemic for onchocerciasis.
Figure B with the courtesy of Rachel Bronzan, created in ArcGIS (https://www.esri.com/en-us/arcgis/products/arcgis-desktop/resources).
Base layers of the map: https://data.humdata.org/dataset/cod-ab-tgo.
Shape files are in " tgo_adm_inseed_itos_20210107_SHP.zip".

Entomology

We geospatially mapped the locations of each of the collection points for which we had information on the geospatial coordinates using the Annual Transmission Potential (ATP) to define transmission intensity (**Fig C**).

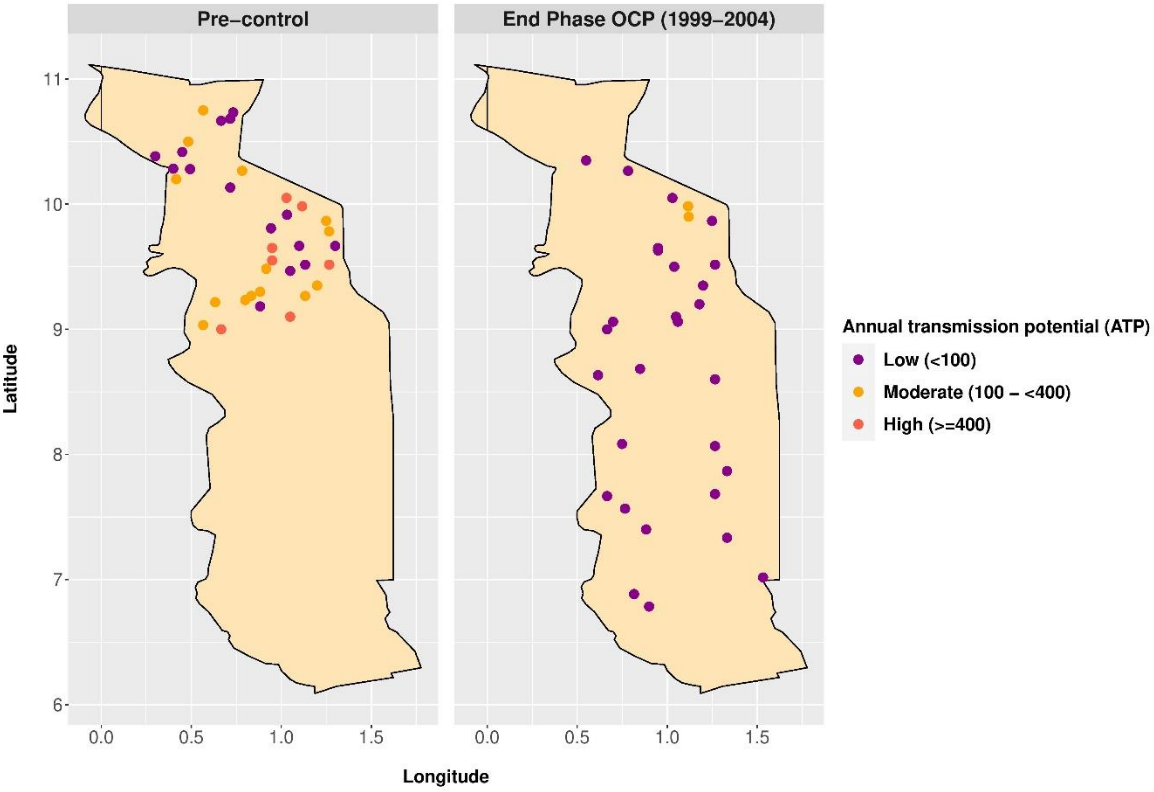

Fig C. Map of the Republic of Togo with measured Annual Transmission Potential per collection point. Annual Transmission Potential (ATP) were classified as low (ATP < 100), moderate (ATP ≥100-<400), or high (ATP≥400). The data points represent the various collection points where entomological surveys were performed. The two panels present data for different time periods: pre-control (left) and during the end phase of OCP (right). Data are not available for more recent years (>= 2012). Figure created in Rstudio [10]. We used the Rstudio packages cowplot, gridGraphics, ggmap, maps, and mapdata. The RStudio IDE is available under the GNU Affero General Public License v3 (Free Software).

Epidemiology

Locations of surveyed villages, classified by crude mf prevalence, at different timepoints is depicted in **Fig D**. Before the start of interventions, most locations where meso- or hyper endemic. The crude mf prevalence declined over time to levels <10% in recent years. Only in Kara and Centrale, some villages with prevalences above 30% still remain in the years 2012 through to 2015. In more recent years (2016-2017), highest mf prevalences of >6% were found in the district of Amou (Igbowou and Kpati Kopé).

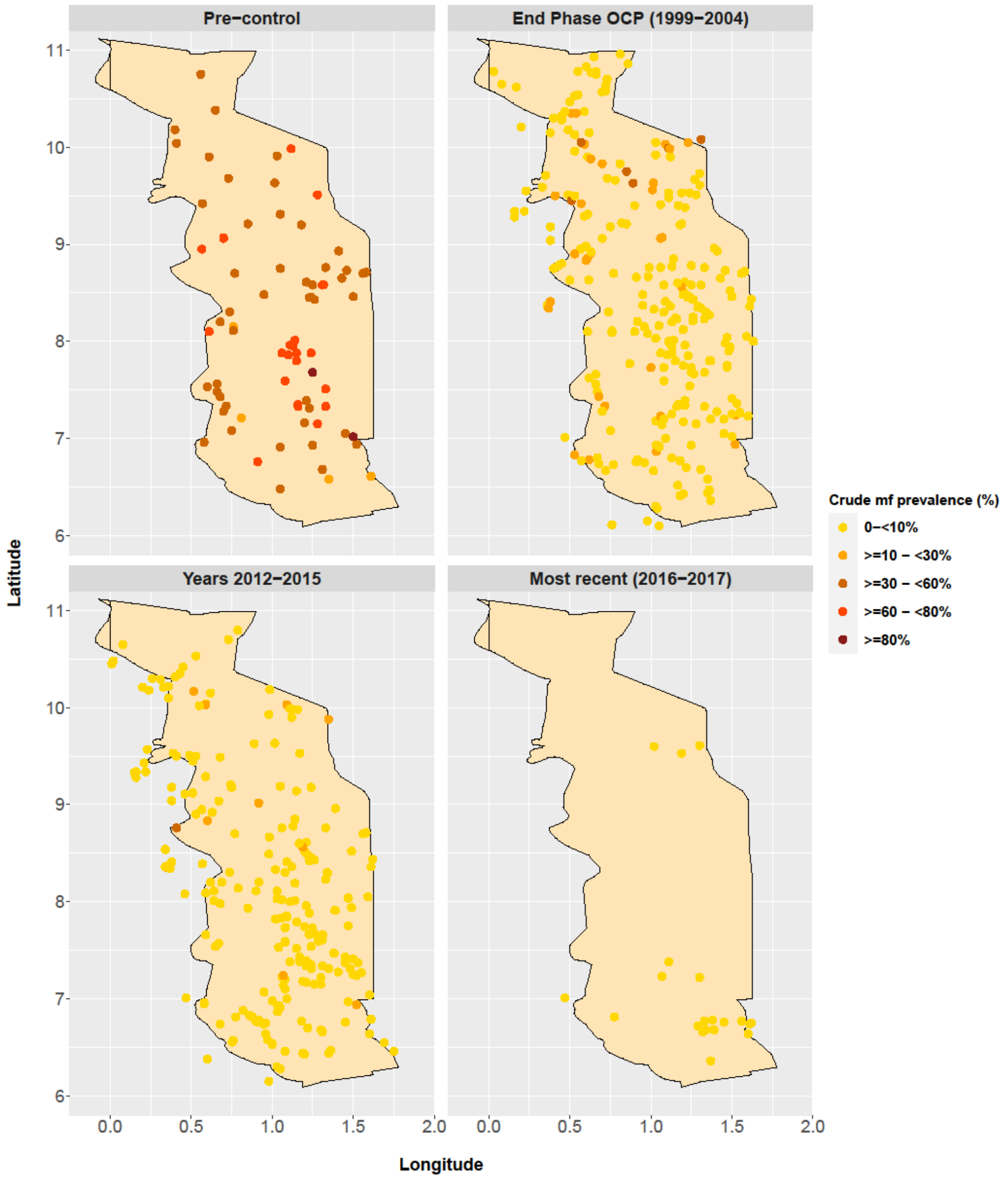

Fig D. Locations of surveyed villages and observed crude mf prevalence levels for four time periods in the Republic of Togo. Crude mf prevalence in percentages as measured through skin snips with one survey per location. There are very little recent mf skin snip results (2016-2017). Figure created in Rstudio [10]. We used the Rstudio packages cowplot, gridGraphics, ggmap, maps, and mapdata. The RStudio IDE is available under the GNU Affero General Public License v3 (Free Software).

Fig E shows the trend in crude mf prevalence by major river basin by data source. The river basins of Mono (no Special Intervention Zone [non-SIZ]), Oti (SIZ), and Volta Lac (some SIZ-villages) seem to show increasing crude mf prevalence since 2010, even after an initial drop around 2005). In the Mono river basin, the tributaries along the rivers of Amou, Anie and Mono were found to have highest crude mf prevalence. Several more recent data with high infection prevalence are reported from a research setting, whereas most impact assessments of the Ministry of Health in Togo of recent evaluation surveys show low infection numbers except in the Kéran, Kpaza and Mô (Pendjari) river basins.

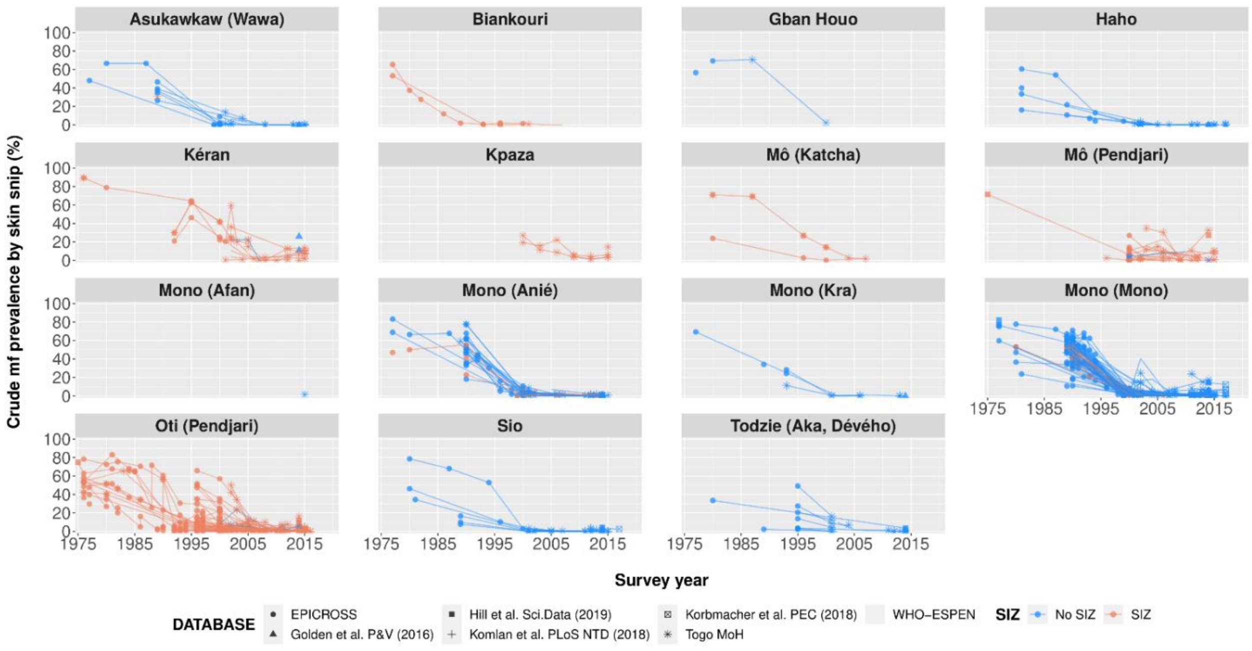

Fig E. Crude mf prevalence (skin snip) over time since 1975 by major river basin (tributary), starting after the initiation of Onchocerciasis Control Programme in 1974 in Togo. The dots represent the reported crude mf prevalence per village, and the lines interconnect the same villages over various survey years. The various symbols represent the different data sources consulted, and the colours represent the location of the surveyed villages in SIZ (red) versus non-SIZ (blue) areas. Figure created in Rstudio [10]. We used the Rstudio packages ggplot2, data.table. The RStudio IDE is available under the GNU Affero General Public License v3 (Free Software).

In **Fig F-J**, we present trends in crude mf prevalence by district within each region. The Savanes region (most northern region) was all designated as SIZ, with most surveys performed in the districts of Kpendjal and Oti (**Fig F**). There were still some villages in the Oti district of the region Savanes with crude mf prevalences above 5%, with one village even reaching 12.5% crude mf prevalence by 2014 even though recent CMFL values are very low (<0.5). In 2011, five villages in the district of Kpendjal were epidemiologically surveyed, and a crude mf prevalence ranging between 0 to 7.5% was found (village Pancerys). Four years later (2015), the mf prevalence declined nicely to 2.9% in the village of Pancerys. Unfortunately, we do not have any follow-up survey data since 2015 from this village. The crude mf prevalences from two other villages in

Kpendjal were 0.8% and 1.52% in 2015. In the latter village, the CMFL was measured at 0.05 only.

In the region of Kara, crude mf prevalence declined satisfactory in most districts (**Fig G**). The whole region of Kara was designated as SIZ, but in four out of six endemic districts the crude mf prevalence was all below 2% in the latest survey. Only in the districts of Bassar and Kéran, relatively high mf prevalences were still observed during the 2014/2015 surveys. In Bassar, mf prevalences ranged between 0.0% to 27.0% with highest mf prevalences measured in the villages along the Mô river. The mf prevalances in the Kéran district ranged from 0.0% to 13.6% during the surveys performed in 2014 and 2015, with highest mf prevalences measured along the Kéran river (more precise, along the Tchitchira collection point). Here, the mf prevalences even seems to have increased again after an initial drop to very low levels in 2008-2010.

The region of Centrale consists of areas that were designated as SIZ and areas where onchocerciasis was considered to be effectively controlled (non-SIZ) (**Fig H**). Indeed, we see that in the districts of Blitta and Tchamba (non-SIZ) as well as Tchaoudjo (SIZ) the onchocerciasis prevalence has reached zero levels in almost all villages (highest prevalence was measured in one village along the Ogou river with a prevalence of 3.5% in 2013 in the district of Tchamba). Only the results across the SIZ-district of Sotouboua are less satisfactory with in five villages a crude mf prevalence of above 5%, reaching a crude mf prevalence of 32.7% in one village along the Mô river.

In the region of Plateaux, the trends in onchocerciais prevalence were very satisfactory in most districts, including in some villages with very high pre-control mf prevalence, and from 2010 onward the mf prevalence was below 5% in most villages (**Fig I**). Some villages in the districts of Amou and Haho still reported mf prevalences of >5%, even reaching 17.5% in surveys performed between 2013 and 2017.

In the region of Maritime, few villages were followed over time on onchocerciasis trends, but mf prevalence has been very low in recent years (**Fig J**).

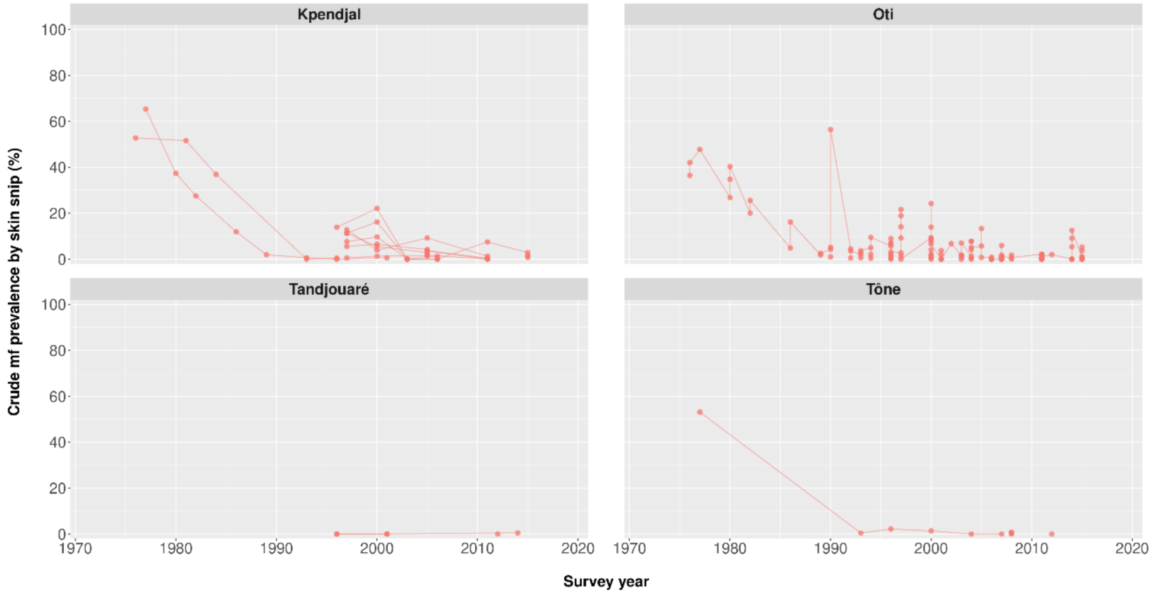

Fig F. Crude mf prevalence (skin snip) over time since 1975 by district in the region of Savanes, starting after the initiation of Onchocerciasis Control Programme in 1974 in Togo. The dots represent the reported crude mf prevalence per village, and the lines interconnect the same villages over various survey years. The region of Savanes was designated in its totality as SIZ (red). We used the Rstudio packages ggplot2, data.table. The RStudio IDE is available under the GNU Affero General Public License v3 (Free Software).

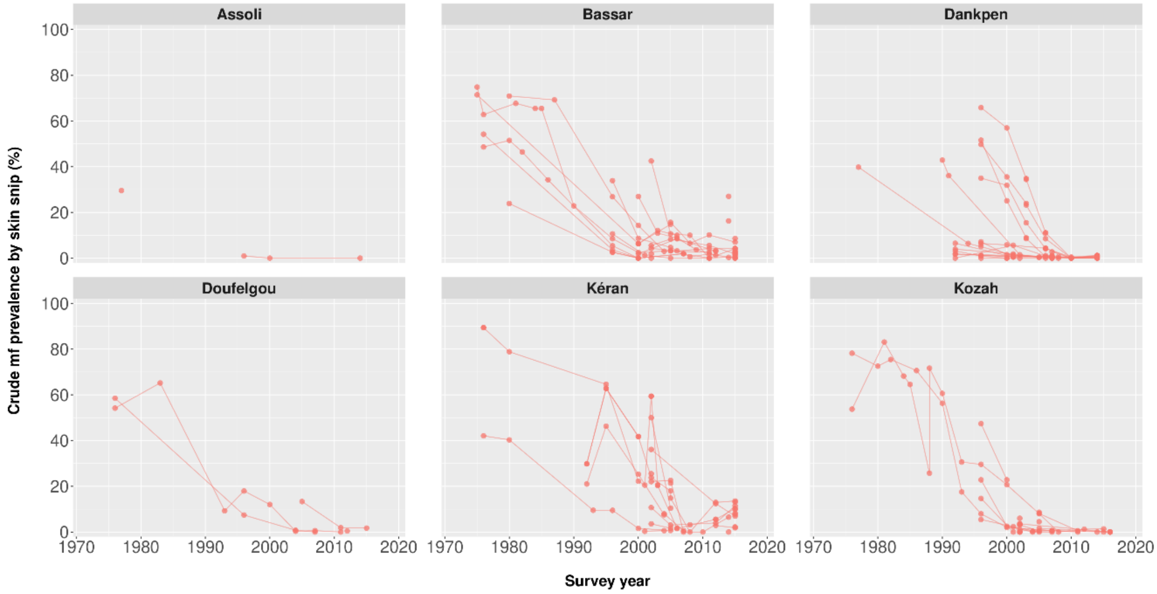

Fig G. Crude mf prevalence (skin snip) over time since 1975 by district in the region of Kara, starting after the initiation of Onchocerciasis Control Programme in 1974 in Togo. The dots represent the reported crude mf prevalence per village, and the lines interconnect the same villages over various survey years. The region of Kara was designated in its totality as SIZ (red). We used the Rstudio packages ggplot2, data.table. The RStudio IDE is available under the GNU Affero General Public License v3 (Free Software).

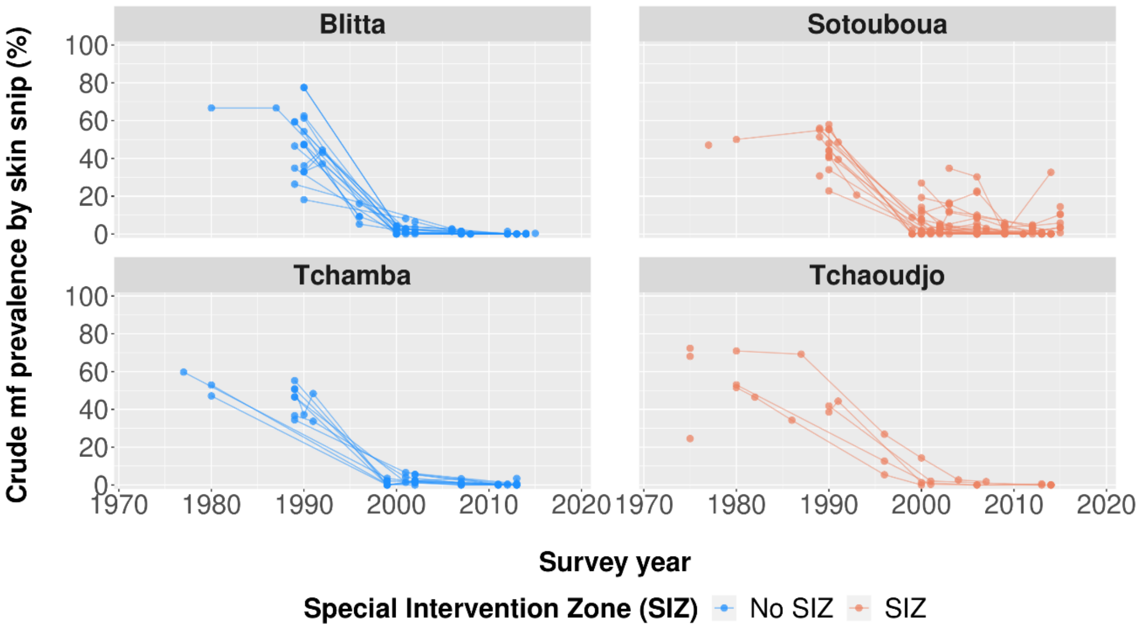

Fig H. Crude mf prevalence (skin snip) over time since 1975 by district in the region of Centrale, starting after the initiation of Onchocerciasis Control Programme in 1974 in Togo. The dots represent the reported crude mf prevalence per village, and the lines interconnect the same villages over various survey years. The various colours represent the location of the surveyed villages in SIZ (red) versus non-SIZ (blue) areas. We used the Rstudio packages ggplot2, data.table. The RStudio IDE is available under the GNU Affero General Public License v3 (Free Software).

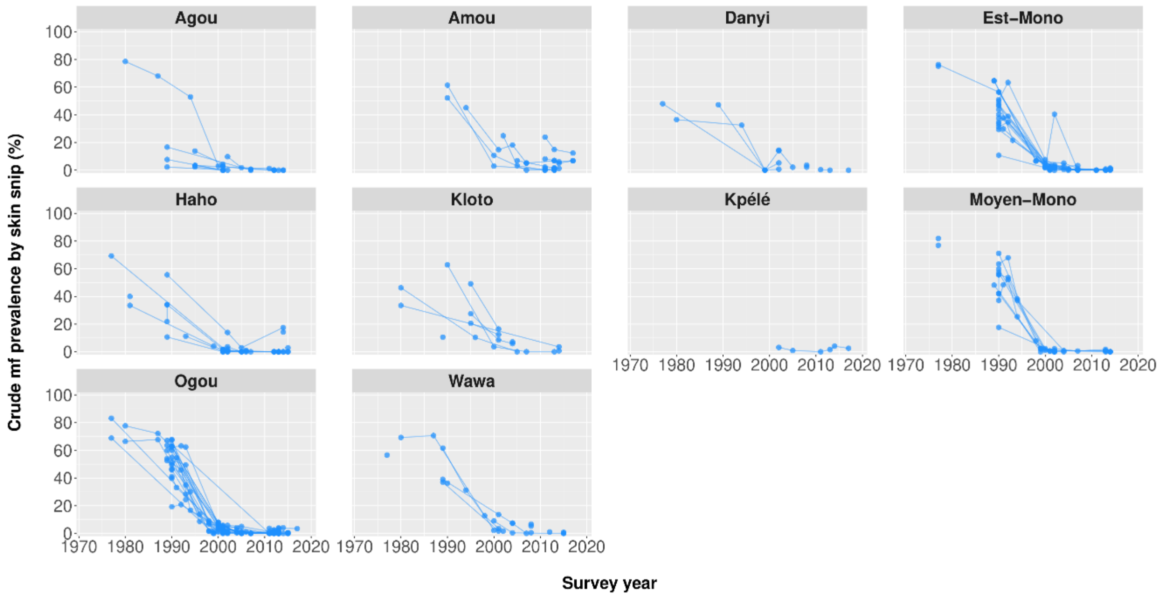

Fig I. Crude mf prevalence (skin snip) over time since 1975 by district in the region of Plateaux, starting after the initiation of Onchocerciasis Control Programme in 1974 in Togo. The dots represent the reported crude mf prevalence per village, and the lines interconnect the same villages over various survey years. The region of Plateaux was not considered to be SIZ (blue). We used the Rstudio packages ggplot2, data.table. The RStudio IDE is available under the GNU Affero General Public License v3 (Free Software).

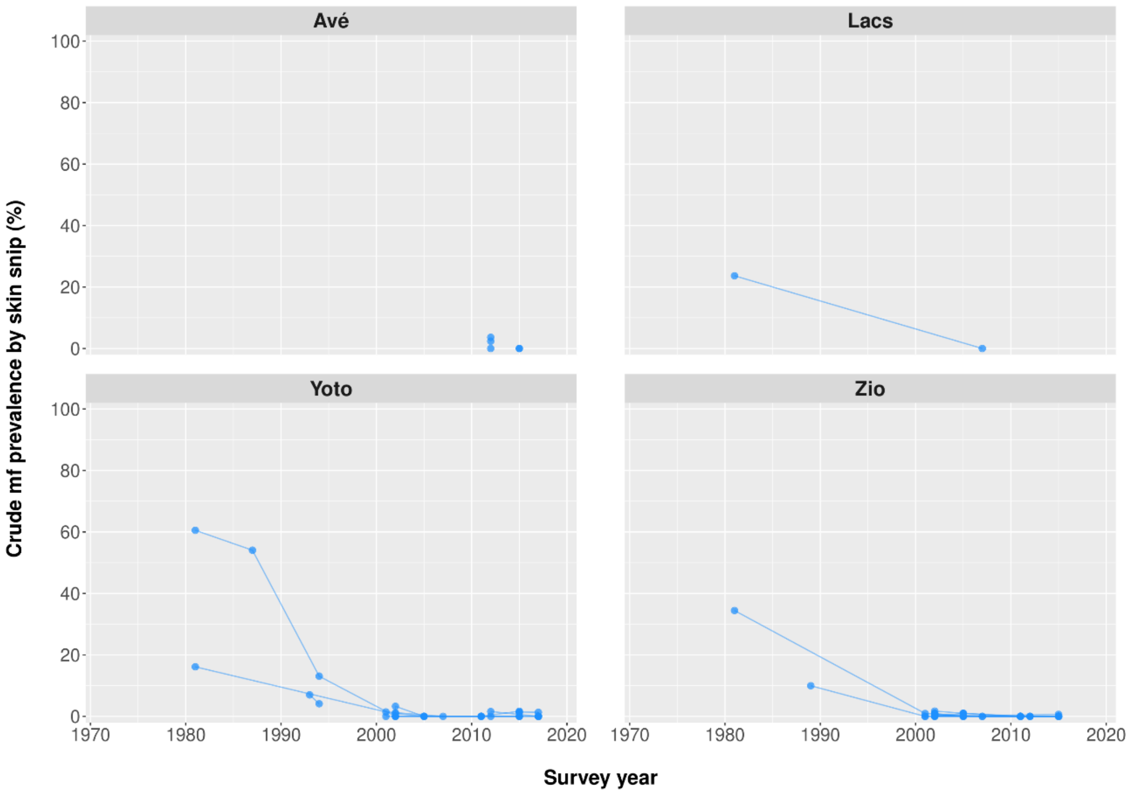

Fig J. Crude mf prevalence (skin snip) over time since 1975 by district in the region of Maritime, starting after the initiation of Onchocerciasis Control Programme in 1974 in Togo. The dots represent the reported crude mf prevalence per village, and the lines interconnect the same villages over various survey years. The region of Maritime was not considered to be SIZ (blue). We used the Rstudio packages ggplot2, data.table. The RStudio IDE is available under the GNU Affero General Public License v3 (Free Software).

Additional OV16 results

The mean prevalence of anti-OV16 antibody positivity against OV16 by region, district, and age group is presented in **Fig K** for data collected between 2014-2017. The data show promising results among the <10 years of age group, particularly when using the commercial RDT as diagnostic test. Higher OV16 prevalences are reported among <10-year-old when using ELISA, particularly in the regions of Kara and Savanes (former SIZ-areas). In addition, **Fig L** shows the size of the sample size of each of the aggregated OV16 results in communities, as stratified by district and region. The figure shows that most OV16 results among 5–10-year-old children using the commercial RDT had quite large sample sizes (>201 people per survey within community), whereas the prototype RDT among individuals <20 years old generally had a low sample size (≤10 individuals per community).

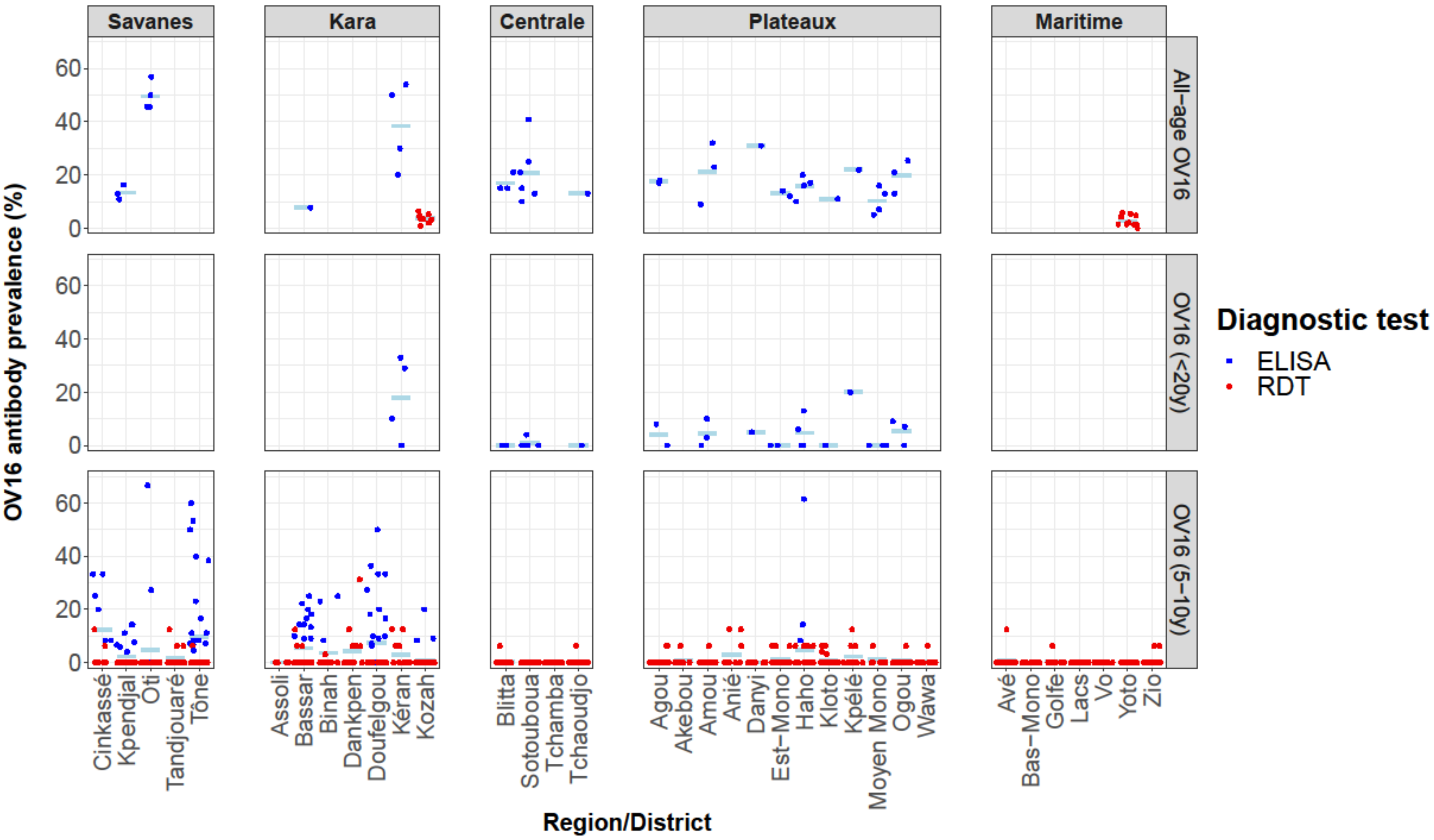

Fig K. Prevalence of anti-OV16 antibody positivity by region, district, and age group. Each dot presents the prevalence observed in a community and dots are coloured according to the diagnostic test that was used; the light blue line shows the mean of village level observations. Data were collected between 2014 and 2017. A random jitter was added to optimise the visualisation of the individual dots, e.g., in the upper panel for Maritime. We used the Rstudio packages ggplot2, data.table. The RStudio IDE is available under the GNU Affero General Public License v3 (Free Software).

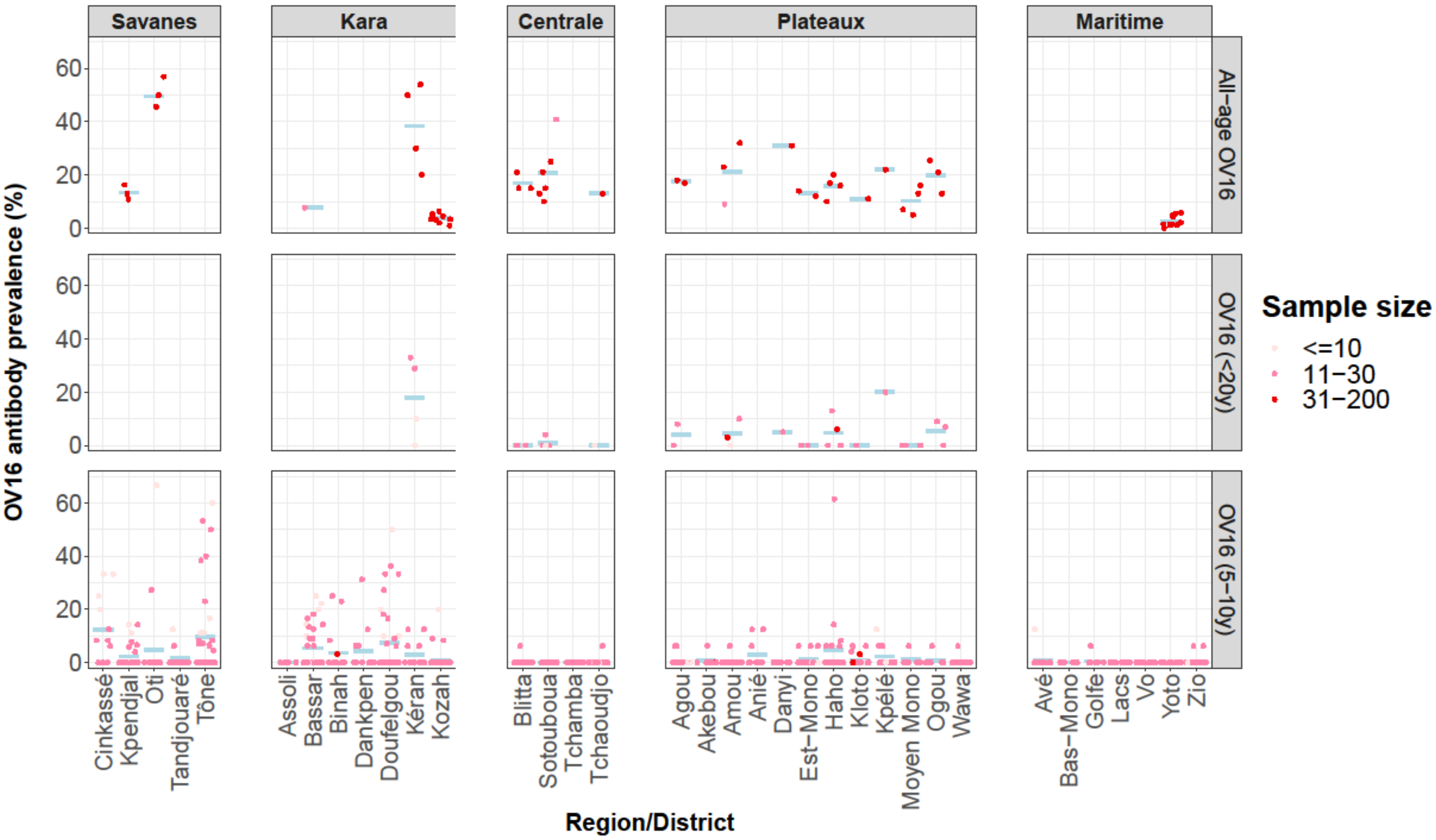

Fig L. Sample size and prevalence of anti-OV16 antibody positivity by region, district, and age group. Each dot presents the prevalence observed in a community and dots are coloured according to the sample size of the study; the light blue line shows the mean of village level observations. Data were collected between 2014 and 2017. A random jitter was added to optimise the visualisation of the individual dots, e.g., in the upper panel for Maritime. We used the Rstudio packages ggplot2, data.table. The RStudio IDE is available under the GNU Affero General Public License v3 (Free Software).

Results of IgG4 or IgG1 responses to O. volvulus antigen (OvAg)

Our database also includes 20 records presenting responsiveness against an adult (crude) worm antigen extract from male and female *O. volvulus* (OvAg). The IgG1 and IgG4 responses of study population can be investigated using an antigen extract of adult *O. volvulus* [11], which is different than testing for presence of antibodies to OV16 antigens (OV16). Nine surveys were performed in 2017 across the Kéran, Mô, and Ôti (former SIZ areas) with OvAg in individuals of <15 years ranging between 28.6% and 83.3% [12]. Highest mean IgG4 seroprevalences to OvAg in children were found in Kéran (51.7%), Mô (23.5%) and 5.2% (Ôti). The IgG4-OvAg ELISA as measured by Komlan and colleagues [13] ranged from 9.4% to 77.8% positivity among children ≤10 years across 11 communities surveyed in 2015, with a mean overall IgG4-OvAg ELISA prevalence of 29.1% across the same three river basins (Kéran, Mô, Ôti) [13]. The study by Komlan et al. [13] reports sensitivities of OvAg- and OV16-specific IgG4-ELISAs to detect Mf-positive individuals of 89.2% and 71.4% respectively. A recently published study demonstrated

that OvAg-specific IgG1 and IgG4 responses among adults were nicely diminished after 30 years post-initiation of MDA to levels observed in endemic *O. volvulus* microfilariae-negative controls across three rural villages in the Centrale region [11].

References

1. Moreau J.P., Prost A., Prod'hon J. Essai de normalisation de la méthodologie des enquêtes clinico-parasitologiques sur l'onchocercose en Afrique de l'Ouest - fdi:09284 - Horizon. Médecine Tropicale. 1978;38: 43–51.
2. World Health Organization. Guidelines for stopping mass drug administration and verifying elimination of human onchocerciasis: Criteria and Procedures. World Health Organization, WHO/HTM/NTD/PCT/20161 Geneva, Switzerland. 2016.
3. Remme J, Zongo JB. Demographic aspects of the epidemiology and control of onchocerciasis in West Africa. Demography and vector-borne diseases. 1989. In: The epidemiology and control of onchocerciasis in West-Africa [Available at: http://hdl.handle.net/1765/50918].
4. World Health Organization. Epidemiology of onchocerciasis: report of a WHO Expert Committee. WHO Technical Report Series. 1976;597.
5. ESPEN Portal. Onchocerciasis maps. Expanded Special Project for Elimination of Neglected Tropical Diseases (ESPEN) World Health Organization Regional Office for Africa [Available at: https://espen.afro.who.int/; Assessed on: 11 May 2023].
6. World Health Organization. Entomological manual for onchocerciasis elimination programmes. World Health Organization. ISBN 978-92-4-006861-2. 2023.
7. Borsboom GJ, Boatin BA, Nagelkerke NJ, Agoua H, Akpoboua KL, Alley EW, et al. Impact of ivermectin on onchocerciasis transmission: assessing the empirical evidence that repeated ivermectin mass treatments may lead to elimination/eradication in West-Africa. Filaria J. 2003;2: 8.
8. World Health Organization. Onchocerciasis and its control. Report of a WHO Expert Committee on Onchocerciasis Control. World Health Organ Tech Rep Ser. 1995;852: 1–104.
9. Albers A, Esum ME, Tendongfor N, Enyong P, Klarmann U, Wanji S, et al. Retarded Onchocerca volvulus L1 to L3 larval development in the Simulium damnosum vector after anti-wolbachial treatment of the human host. Parasit Vectors. 2012;5: 1–10.
10. RStudio Team (2020). RStudio: Integrated Development for R. RStudio, PBC, Boston, MA [Available at: http://www.rstudio.com/; Accessed on: 18 January 2024].
11. Johanns SI, Gantin RG, Wangala B, Komlan K, Halatoko WA, Banla M, et al. *Onchocerca volvulus*-specific antibody and cellular responses in onchocerciasis patients treated

annually with ivermectin for 30 years and exposed to parasite transmission in central Togo. PLoS Negl Trop Dis. 2022;16: e0010340.

12. Korbmacher F, Komlan K, Gantin RG, Poutouli WP, Padjoudoum K, Karabou P, et al. *Mansonella perstans*, *Onchocerca volvulus* and *Strongyloides stercoralis* infections in rural populations in central and southern Togo. Parasite Epidemiol Control. 2018;3: 77–87.
13. Komlan K, Vossberg PS, Gantin RG, Solim T, Korbmacher F, Banla M, et al. *Onchocerca volvulus* infection and serological prevalence, ocular onchocerciasis and parasite transmission in northern and central Togo after decades *of Simulium damnosum s.l.* vector control and mass drug administration of ivermectin. Freeman MC, editor. PLoS Negl Trop Dis. 2018;12: e0006312.
