# Supplementary material for: Impact of ivermectin and vector control on onchocerciasis transmission in Togo: Assessing the empirical evidence on trends in infection and entomological indicators
Source: PLoS Negl Trop Dis. 2024 Jul 22;18(7):e0012312. doi: 10.1371/journal.pntd.0012312 (PMC11293710; doi:10.1371/journal.pntd.0012312)
Supplement: S2 Text — (PDF) [file pntd.0012312.s003.pdf]

# S2 Text: Explanation of variables of the databases

Supplement to:

## Impact of ivermectin and vector control on onchocerciasis transmission in Togo: assessing the empirical evidence on trends in infection and entomological indicators.


**Authors:**

Natalie V.S. Vinkeles Melchers[1,2#], Sibabi Agoro[3], Kwamy Togbey[3], Koffi Padjoudoum[3], Ibrahim Gado Telou[3], Potchoziou Karabou[3], Touka Djatho[3], Michel Datagni[3], Ameyo Monique Dorkenoo[4], Yao Kassankogno[5]†, Rachel Bronzan[6], Wilma A. Stolk[2]

**Author affiliation:**

1. Health & Society Group, Social Sciences Department, Wageningen University & Research, Wageningen, The Netherlands.
2. Department of Public Health, Erasmus MC, University Medical Center Rotterdam, Rotterdam, The Netherlands.
3. National Institute of Hygiene, Ministry of Health, Public Hygiene and Universal Access to Care, Lomé, Togo.
4. Faculté Des Sciences de La Santé, Université de Lomé, Boulevard Eyadema, 01BP 1515, Lomé, Togo.
5. Health and Development International (HDI), Lomé, Togo.
6. Bill & Melinda Gates Foundation, 500 5th Ave N, Seattle, WA 98109, United States of America.

**Correspondence to:**

Natalie V.S. Vinkeles Melchers, PhD. MSc. MPH.

Health & Society Group, Social Sciences Department, Wageningen University & Research, Hollandseweg 1, 6706 KN Wageningen, The Netherlands.

**Email:** natalie.vinkelesmelchers@wur.nl

† Deceased.

The following document describes the variables used in the six tab-pages of the **S1 Database**. Please therefore read the following document for a detailed description of the tab-pages and variables.

This Excel file consists of six tab-pages:

1. Skin snip (mf) prevalence
2. OV16 prevalence
3. Prevalence of other diagnostic measures (e.g., palpable nodules)
4. Entomological database including vector control, ABR, ATP
5. MDA history at the village-level
6. MDA history at the district-level

## 1. Skin snip (mf) prevalence

We used the EPICROSS (historical OCP epidemiological database) as backbone for this database (first tab, "1. Skin snip"). Many of the variable names and codes remain the same. We hereby provide a variable list.

| | |
|---|---|
| VILCODE | Unique codes of surveyed villages as provided by EPICROSS. |
| VILNOM | Village names of surveyed villages. |
| VILPAY | Unique code of the OCP country (here: Togo). |
| PAYNOM | Name of the OCP country. |
| BAS_CODE | Unique code of the tributaries of river basins. |
| BASNOM | Names of the tributaries of river basins. |
| SIZ | Whether a village was classified within an area designated as Special Intervention Zone (SIZ). This variable was added for analysis purposes. |
| VECTOR_START | Start year of vector control |
| VECTOR_END | End year of vector control |
| GBA_CODE | Unique code of the major of river basin. |
| GBALIB | Name of the major of river basin. |
| PHASE_CODE | Unique code designated by OCP to the various phases of implementation of interventions. |
| PHANOM | Name of the implementation phase of interventions, as designated by OCP. |
| DIS_CODE | Unique code of a respective district in which the village is located. |
| DISNOM | Name of the district (ADMIN2) in which the village is located. |
| REG_CODE | Unique code of a respective region in which the village is located. |
| REGNOM | Name of the region (ADMIN1) in which the village is located. |
| PC_CODE | Unique code of a respective entomological collection point. |
| PCLIBE | Name of the entomological collection point. |
| LAT.DD. | Latitude decimal degrees (DD) of the surveyed village. |
| LON.DD. | Longitude decimal degrees (DD) of the surveyed village. |
| Survey_year | Year of the epidemiological survey performed in the village. |
| REC_BASE | Number of people inhabiting a respective village according to the census performed at the time of the survey. |
| PRE_BASE | Number of people present in a respective village at the time of the survey. |
| EXA | Number of people examined during an epidemiological survey. |
| POS_BASE | Number of people positive by skin snip. |
| PREV_cr | Crude mf prevalence (POS_BASE $\div$ EXA $\times$ 100) |

| | |
|---|---|
| PREV_st | Standardised mf prevalence according to age- and sex standardisation of the OCP. |
| CMFL | Community microfilarial load |
| CECI | Cécité, prevalence of vision loss (visual impairment and blindness), as measured during OCP surveys. This measure was not collated during this study. |
| DIAGNOSIS | Type of diagnosis of prevalence measures. This variable was added for analysis purposes. |
| DATABASE | The original database of the data. The references are listed under the 'References' heading. |
| Pre.control | Classification of data as pre-control (before initiation of vector control or MDA). This variable was added for analysis purposes. |
| Period | Classification of data into three time periods (pre-control, end phase OCP, recent). If the record fell outside one the periods, the data points were not included in the mapping exercise. This variable was added for analysis purposes. |
| Endem_class | Classification of villages into five endemicity strata based on the pre-control mf prevalence (sporadic, hypoendemic, mesoendemic, hyperendemic, very hyperendemic). |

## 2. OV16 prevalence

The variables in this database corresponds to the skin snip prevalence database (first tab, "1. Skin snip"). The variables that differ from the aforementioned list of variables, will be described here.

| | |
|---|---|
| EXA | Number of people examined during an epidemiological survey. |
| POS | Number of people positive by OV16. |
| PREV | Prevalence of the people tested positive by OV16 as compared to all people examined (POS ÷ EXA × 100). |
| Lowerbound | Lower bound of the 95% confidence interval around the prevalence of OV16 positive individuals in a respective village, as measured by the two-sided binomial test. |
| Upperbound | Upper bound of the 95% confidence interval around the proportion of OV16 positive individuals in a respective village, as measured by the two-sided binomial test. |

| | |
|---|---|
| DIAGNOSIS | "OV16 (5-10y)" (OV16 measured in children of ≤10 years old), or "OV16 (<20y)" (OV16 measured in children and young adults up to 20 years of age), or "All-age OV16" (OV16 measured in individuals of all ages). |
| Type_test | Diagnostic test for prevalence of antibody against OV16 serology, either Rapid Diagnostic Test (RDT), or enzyme-linked immunosorbent assay (ELISA). |

## 3. Prevalence of other diagnostic measures (e.g., palpable nodules)

The variables in this database are very similar to the skin snip prevalence database (first tab, "1. Skin snip"). The variables that differ from the aforementioned list of variables, will be described here.

| | |
|---|---|
| EXA | Number of people examined during an epidemiological or research-specific survey. |
| POS | Number of people positive by the respective diagnostic tool. |
| PREV | Prevalence as measured by the respective diagnostic tool (POS ÷ EXA × 100) |
| DIAGNOSIS | "MFT" (Membrane filtration technique) or "Nodules" (palpation-based detection of nodules that could be attributed to onchocerciasis with reasonable certainty). |
| Comments | Additional comments relevant to the respective record. |

## 4. Entomological database including vector control

Many variables in this database corresponds to the skin snip prevalence database (first tab, "1. Skin snip"). The variables that differ from the aforementioned list of variables, will be described here.

| | |
|---|---|
| CODEPC | Unique code of the blackfly collection point, as provided by the OCP. |
| PC_NAME | Name of the entomological collection point. |
| PCLONGC | Longitude decimal degrees (DD) of the blackfly collection point. |
| PCLATIC | Latitude decimal degrees (DD) of the blackfly collection point. |
| Riverbasin | A stratification of the large river basins into the main and commonly used names of the endemic river basins in Togo. This variable was added for analysis purposes. |
| ANNEE | Year of the entomological survey performed in the blackfly collection point (similar to Survey_year in database 1). |

| | |
|---|---|
| ABR | Annual Biting Rate, please be referred to Text S1 for definitions. |
| ATP | Annual Transmission Potential, please be referred to Text S1 for definitions. |
| ATPCOR | Corrected Annual Transmission Potential, ATP corrected for *Onchocerca ochengi* (i.e., non-pathogenic parasite species to humans). |
| NBJRS | "Nombre des jours", Number of days that blackflies were collected at the collection point. |
| NBMOIS | "Nombre des mois", Number of months that blackflies were collected at the collection point. |
| Flies_exa | Number of blackflies examined from the blackflies collected at the respective collection point. Information only provided for more recent studies. Variable added for the purpose of this study. |
| Flies_pos | Number of blackflies positive from the blackflies collected and examined at the respective collection point. Information only provided for more recent studies. Variable added for the purpose of this study. |
| Prev_cr_flies_pos.1000 | Number of positive blackflies out of the total number of blackflies examined per a 1,000 flies from the respective collection point (Flies_pos ÷ Flies_exa × 1,000). Information only provided for more recent studies. Variable added for the purpose of this study. |
| Infec_F.1000parousF | Number of positive female blackflies out of the total number of parous female blackflies examined per a 1,000 flies from the respective collection point. Information only provided for more recent studies. Variable added for the purpose of this study. |

## 5. MDA history at the village-level

Many variables in this database corresponds to the skin snip prevalence database (first tab, "1. Skin snip"). The variables that differ from the aforementioned list of variables, will be described here.

| | |
|---|---|
| FOSA/MSTR | Health district (ADMIN3) where ivermectin Mass Drug Administration (MDA) may have been distributed from. |
| Mois | Month that the MDA round took place in the respective village. |
| Recenses | Number of people present/inhabiting a respective village according to the census (similar to REC_BASE) performed at the time of the survey. |
| Traites | Number of treated by ivermectin MDA within a respective village at the time of the survey. |

| | |
|---|---|
| TheraCov | Therapeutic coverage of ivermectin MDA of the respective village, calculated as (Traites ÷ Recenses × 100). |
| Sourcefile | Specific source file among the various excel files obtained from the Togo Ministry of Health. |

## 6. MDA history at the district-level

We used the ESPEN (https://espen.afro.who.int/) as backbone for this database. Most of the variable names and codes remain the same, and can be found on the ESPEN portal [1]. We added mean district-level therapeutic coverage of ivermectin MDA from other databases than ESPEN to this database for completeness (EpiCov). We only explain variables additionally added or so far unclear, for analysis purposes.

| | |
|---|---|
| MDA_freq | Ivermectin MDA frequency per annum in the respective district (ADMIN2, Implementation Unit). |
| Cum_MDA | Cumulative number of MDA, as counted by the number of years that MDA is provided in the district. |
| Cum_MDA_rounds | Cumulative number of MDA, as counted by the number of MDA rounds provided in the district (taking account of multiple MDA rounds per year). |
| EffMDA | Coding for district and year when effective MDA was provided, as defined as therapeutic coverage ≥65% (0 = not effective; 1 = effective). |
| EpiCov | The district-level therapeutic coverage of ivermectin MDA, measured as the population treated over the population requiring treatment within a district (PopTreat ÷ PopReq × 100). For other sources that ESPEN, we calculated the mean district-level therapeutic coverage if population numbers were missing. |
| Source_thera_cov | The data source (database) from where we collated the records used to estimate the district-level therapeutic coverage of MDA. |
| MDA_geo_cov | The district-level geographic coverage of ivermectin MDA, measured as the number of villages treated out of the total villages eligible for treatment within a district. |
| Source_geo_cov | The data source (database) from where we collated the records used to estimate the district-level geographic coverage of MDA across eligible villages. |
| Comments | The ESPEN database, as downloaded on 11$^{th}$ of May 2021, contained multiple records with unrealistic therapeutic coverages of MDA (>100%), |

## References

1. ESPEN Portal. Onchocerciasis maps. Expanded Special Project for Elimination of Neglected Tropical Diseases (ESPEN). World Health Organization Regional Office for Africa [Available at: https//espen.afro.who.int/].
